# Mechanistic Mathematical Modeling Tests Hypotheses of the Neurovascular Coupling in fMRI

**DOI:** 10.1371/journal.pcbi.1004971

**Published:** 2016-06-16

**Authors:** Karin Lundengård, Gunnar Cedersund, Sebastian Sten, Felix Leong, Alexander Smedberg, Fredrik Elinder, Maria Engström

**Affiliations:** 1 Department of Medical and Health Sciences, Linköping University, Linköping, Sweden; 2 Center for Medical Image Science and Visualization (CMIV), Linköping University, Linköping, Sweden; 3 Department of Biomedical Engineering, Linköping University, Linköping, Sweden; 4 Department of Clinical and Experimental Medicine, Linköping University, Linköping, Sweden; University College London, UNITED KINGDOM

## Abstract

Functional magnetic resonance imaging (fMRI) measures brain activity by detecting the blood-oxygen-level dependent (BOLD) response to neural activity. The BOLD response depends on the neurovascular coupling, which connects cerebral blood flow, cerebral blood volume, and deoxyhemoglobin level to neuronal activity. The exact mechanisms behind this neurovascular coupling are not yet fully investigated. There are at least three different ways in which these mechanisms are being discussed. Firstly, mathematical models involving the so-called Balloon model describes the relation between oxygen metabolism, cerebral blood volume, and cerebral blood flow. However, the Balloon model does not describe cellular and biochemical mechanisms. Secondly, the metabolic feedback hypothesis, which is based on experimental findings on metabolism associated with brain activation, and thirdly, the neurotransmitter feed-forward hypothesis which describes intracellular pathways leading to vasoactive substance release. Both the metabolic feedback and the neurotransmitter feed-forward hypotheses have been extensively studied, but only experimentally. These two hypotheses have never been implemented as mathematical models. Here we investigate these two hypotheses by mechanistic mathematical modeling using a systems biology approach; these methods have been used in biological research for many years but never been applied to the BOLD response in fMRI. In the current work, model structures describing the metabolic feedback and the neurotransmitter feed-forward hypotheses were applied to measured BOLD responses in the visual cortex of 12 healthy volunteers. Evaluating each hypothesis separately shows that neither hypothesis alone can describe the data in a biologically plausible way. However, by adding metabolism to the neurotransmitter feed-forward model structure, we obtained a new model structure which is able to fit the estimation data and successfully predict new, independent validation data. These results open the door to a new type of fMRI analysis that more accurately reflects the true neuronal activity.

## Author Summary

Functional magnetic resonance imaging (fMRI) is a widely used technique for measuring brain activity. However, the signal registered by fMRI is not a direct measurement of the neuronal activity in the brain, but it is influenced by the interplay between the metabolism, blood flow and blood volume in the active area. This signal is called the blood-oxygen-level dependent (BOLD) response and occurs when the blood supply to the active area increases in response to neuronal activity. The mechanisms that the cells use to influence the blood supply are not fully known, and therefore it is difficult to know the true neuronal signalling only from inspection of the fMRI signal. In this article, we present a new mathematical model built on the physiological mechanisms thought to underlie the BOLD response. We could successfully fit the model to data and predict the activity caused by new stimuli. By using the validated model we investigated physiological mechanisms that cause different parts of the BOLD response.

## Introduction

Functional magnetic resonance imaging (fMRI) measures brain activity by detecting associated changes in blood oxygenation through the blood-oxygen-level dependent (BOLD) response. The BOLD response is caused by time-dependent changes in deoxyhemoglobin (dHb) concentration [1]. Although the BOLD response reflects neuronal activity through the neurovascular coupling [2], the mechanisms causing the response are still not fully understood. Here, we investigate these mechanisms by mathematical modeling using a systems biology approach.

**Fig 1:**
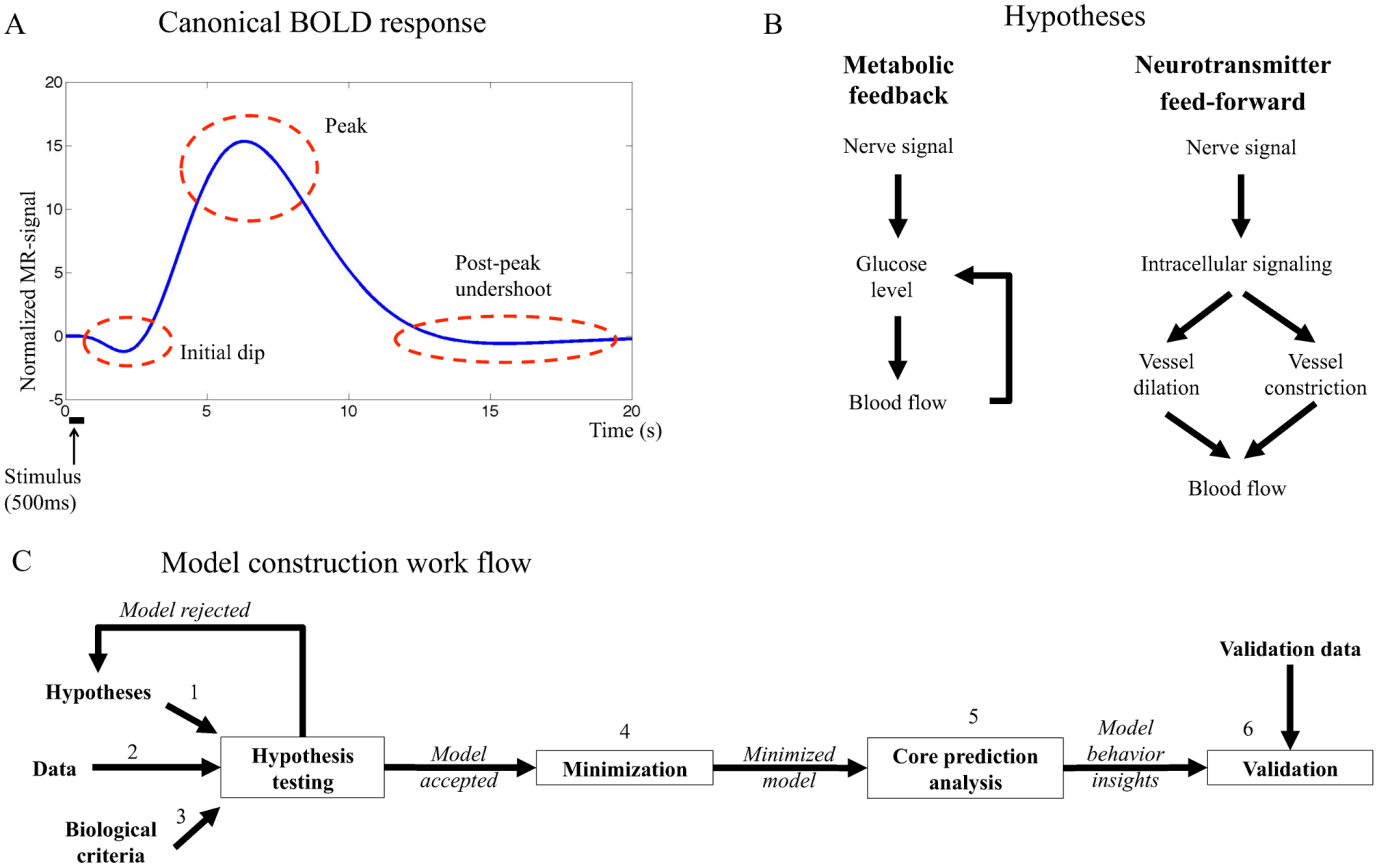
**A. Canonical BOLD response to a brief stimulus**. The initial dip, the peak and the post-peak undershoot are marked in the figure. **B. Main mechanisms of the hypotheses investigated in this work.** The metabolic hypothesis (left) suggests feedback signaling where decreased blood glucose levels trigger cerebral blood flow (CBF) increase, which in turn delivers more glucose to the activated area. The neurotransmitter feed-forward hypothesis (right) suggests feed-forward signaling with two competing arms where the negative arm results in decreased CBF and the positive in increased CBF. The balance between the actions in both arms determines the shape of the BOLD response. **C. The model construction workflow in this article.** The biological hypothesis is translated to equations firmly based in the biological mechanisms (1), this system of equations is the model structure. Data is collected (2) and the model structure is fitted to the data by optimizing the parameters. If the model can not fit the data, the model structure is rejected or altered. If the model can fit the data, it is analyzed and evaluated to control that it does so in a biologically plausible way (3). If the model does not fulfil these criteria, it is rejected. If the model is accepted, the model is minimized in order to identify key mechanisms of the system (4) and core predictions of not yet performed experiments are made (5). In the next step, new data are collected, using experimental setup based on previous predictions, and the model predictions are compared to the new data set (6). If the predictions are satisfactory, the model is accepted. If the predictions fail the model is rejected, and a new iteration of alterations and analyses is performed taking both old and new data into consideration. The model construction workflow can be iterated as many times as needed until a satisfactory model has been reached.

The BOLD response endures approximately 15 seconds after a short neural stimulus and it has several characteristic features (Fig 1A) [3]: (i) During the first couple of seconds a shallow undershoot, referred to as the initial dip, is sometimes observed in activated areas of the brain [4][5]. The initial dip is hypothesized to reflect an increased cerebral metabolic rate of oxygen (CMRO_2_) that is followed by an increase of dHb content in the blood. (ii) At 6–8 s after the stimulus, the BOLD response peaks as a result of increased cerebral blood volume (CBV) and/or increased cerebral blood flow (CBF). (iii) After the peak, the BOLD response decays and shows a post-peak undershoot before returning to baseline. The mechanisms controlling these processes (i-iii) remain unresolved, and there are at least three different approaches to understand these mechanisms.

The first approach is centered around mathematical modeling. One of the most common approaches is to model the hemodynamic response function (HRF) using the so-called Balloon model [6][7][8][9], which has been of paramount importance in the development of fMRI image analysis [10]. The Balloon models describe the interplay between CMRO_2_, CBV, and CBF. The dynamics of these three entities are described in part by purely phenomenological descriptions, such as convolutions with the covariate gamma functions, and in part by physical models *e.g.* describing the dynamics between CBV and CBF in an expanding balloon. In other words, these Balloon models typically do not incorporate intracellular and biochemical mechanisms involved in cell metabolism or intra-cellular signaling processes related to the BOLD response. Nevertheless, there do exist mathematical models that also incorporate intracellular metabolism [11], but ultimately even these models explain the actual BOLD response via the Balloon model, which appears as a sub-model in the complete model. Other models, which are not extensions of the Balloon model, describe *e.g.* spatiotemporal properties of the BOLD response as hemodynamic traveling waves [12] or oxygen transport in the brain by modeling CBF with a linear flow model and CMRO_2_ using a gamma function [13].

The second approach to understanding the BOLD response is centered around the so-called metabolic feedback hypothesis. According to this classical hypothesis (Fig 1B, left), the BOLD response is the result of a tight connection between glucose metabolism and blood flow; when the brain is activated, the neurons consume more energy, resulting in decreased blood glucose and oxygen levels [14][15], which trigger a feedback signal increasing CBF to meet metabolic demands. In other words, the metabolic hypothesis is centered around a feedback control to keep glucose level constant.

The third and final approach relevant to this paper is referred to as the neurotransmitter feed-forward hypothesis. This hypothesis is reviewed in *e.g* [16], and it is today more actively discussed than the metabolic feedback hypothesis. The neurotransmitter feed-forward hypothesis (Fig 1B, right) suggests sequential feed-forward signaling where neurotransmitters, especially glutamate, cause neurons and astrocytes to activate a chain of intracellular events, involving the release of nitric oxide (NO) or arachidonic acid (AA) metabolites, which in turn control constriction and dilation of the blood vessels. In this way, the feed-forward system “anticipates” the increased need, and goes directly from increased neural activity to increased blood supply.

Of the three approaches mentioned above, only the first involves mathematical modeling and these models are focused mainly on the phenomenological description of the HRF. However, although the metabolic feedback and the neurotransmitter feed-forward hypotheses have been extensively studied through purely experimental approaches, these two hypotheses have never been implemented as mathematical models. It is therefore not known whether the proposed mechanisms of the metabolic feedback and the neurotransmitter feed-forward hypotheses actually would produce a BOLD response or not.

Model-based testing of mechanistic hypotheses has been done in biological research for many years, and has gained increased interest through the rise of systems biology. As mentioned above, mathematical models are already standard when analyzing fMRI, but these models are partially phenomenological and have not been developed to test intracellularly centered hypotheses such as the metabolic feedback and the neurotransmitter feed-forward hypotheses. In contrast, intracellular mechanistic models are the main focus in systems biology, and here hypotheses are formulated as direct representations of the assumed biochemical reactions [17][18]. This formulation allows for a new type of data analysis, which revolves around two steps: (i) rejections and (ii) uniquely identified core predictions (Fig 1C). This model-based approach provides a more comprehensive, correct, and verifiable analysis, compared to analyses based on inspection and reasoning. In other words, while it sometimes may seem logical to draw a certain conclusion based on visual inspection of some given data, we and others have repeatedly shown that such manual inspections often lead to incorrect, or at the very least incomplete, interpretations of the data [19, 20].

In this paper, we provide a first systems biology analysis of the metabolic feedback and the neurotransmitter feed-forward hypotheses (Fig 1B) with regards to their ability to describe the BOLD response. We show that neither of the two hypotheses alone can satisfactorily describe the response. In contrast, a feed-forward mechanism with added oxygen metabolism can provide a satisfactory explanation of the BOLD response measured in fMRI.

## Materials and Methods

### Mechanistic modeling

Mechanistic modeling has within systems biology evolved into an iterative process, which alternates between model-based data analysis and the collection of new experimental data. This process is outlined in Fig 1C. In Step 1, existing hypotheses are reformulated into a set of mathematical equations. In this paper, the two main hypotheses are the metabolic feedback and the neurotransmitter feed-forward hypotheses. In Step 2, data are collected, which in this paper correspond to BOLD responses during visual stimulation. The model is then fitted to the data by optimization of the model parameters. Step 3 involves the formulation of biological criteria which might not be found in the collected data, but which have been previously reported in the literature and which the model must fulfil. Step 1–3 compose the hypothesis testing, which includes formulation, fitting and testing of the models. Model testing investigates which sets of equations can and cannot explain the given data, and whether or not these explanations are biologically realistic. This analysis leads to either rejection, which bring the process back to the model formulation step, or acceptance and further analysis. Step 4, minimization, is a simplification of the model where as many states and parameters as possible are removed in order to identify key mechanisms of the system and facilitate computation and overview of the model. In Step 5, further analysis of the explanations consists of the identification of relevant *core predictions*, i.e. uniquely identified predictions with uncertainty [21]. These predictions may sometimes be suitable for experimental testing, and this leads to collection of new data, Step 6, which in turn leads to the final testing and analysis of the model. Sometimes, interesting model behaviors are discovered in this final step, which might lead back to the hypothesis testing step for further investigation. In this way, systems biology modeling has the potential to be a never-ending cycle, but for each step that is passed, new information about the system is obtained. The iterations end when the model is satisfyingly detailed or no more suitable data can be gathered.

#### Model structures are formulated as ordinary differential equations

The models herein are formulated using ordinary differential equations (ODEs), which have the following general structure

$$\dot{x}=f\left(x,p_{x},u\right)$$ (1)

$$x\left(0\right)=x_{0}$$ (2)

$$\hat{y}=g\left(x,p_{x},p_{y},u\right)$$ (3)

where *x* are the states, describing the concentration or amount of various substances; where $\dot{x}$ represents the derivative of the states with respect to time; where *f* and *g* are non-linear smooth functions; where *p*_*x*_ are the parameters used to calculate *f*, here kinetic rate constants; where *u* is the input, here the visual stimuli given to the subjects; where *x*(0) contains the values of the states at time *t* = 0, and where these values are described by the parameters *x*_0_; where $\hat{y}$ are the simulated model outputs corresponding to the measured experimental signals, here the BOLD response; and where *p*_*y*_ are parameters only appearing in the measurement equations, here scaling parameters. Recall that there are three types of parameters, *p*, with potentially unknown values,

$$p=(p_{x},x_{0},p_{y})$$ (4)

How these parameters are determined and evaluated is described below. Note that *x*, *u*, and *y* depend on *t*, but that the notation is dropped unless the time-dependence needs to be especially stated, as in Eq (2). All symbols in Eqs (1), (2) and (3) are vectors.

In the formulation of mechanistic hypotheses into ODEs, there are three levels, which are distinguished using the following notation. The *hypothesis* *x* is denoted *M*_*x*_, where *x* here has the values *m*, *n*, and *nm*, corresponding to the metabolic feedback, the neurotransmitter feed-forward, and the extended neurotransmitter feed-forward hypothesis hypotheses, respectively. Each of these hypotheses have been implemented using alternative sets of equations, corresponding to further specifications and assumptions, and these alternatives are tagged using additional numbers. Such a set of equations is usually referred to as a *model structure* [22]. Finally, a model structure is referred to as a *model* if a set of specific parameter values has been chosen, and these parameters are specified with a final bracket. For example, $M_{n4}\left({\hat{p}}_{4}\right)$ denotes the 4th model structure implementing the neurotransmitter feed-forward hypothesis, which should be analyzed using the parameters in ${\hat{p}}_{4}$.

#### Model structures

**Fig 2:**
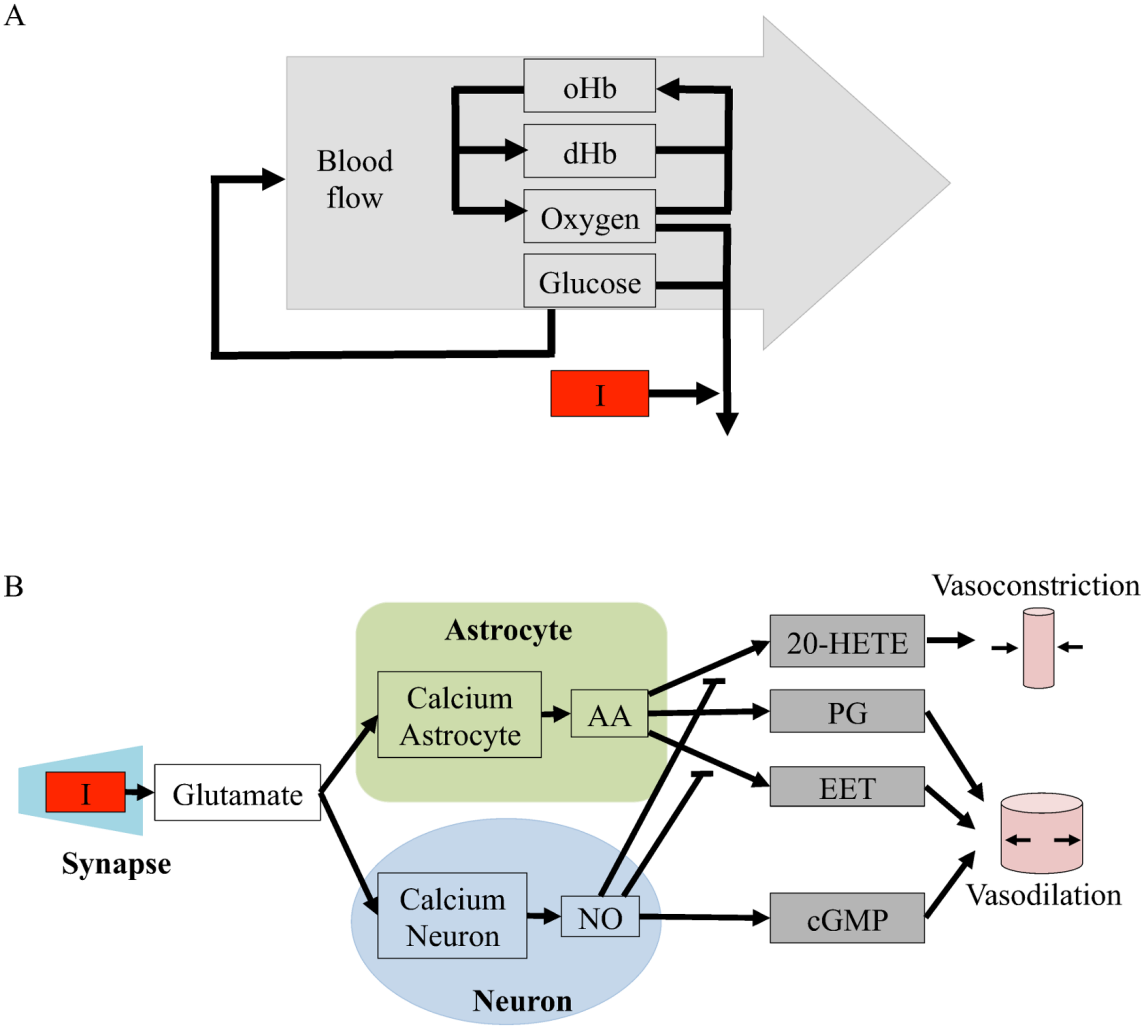
Schematic overviews of the initial model structures evaluated in this paper. I = the stimulus, which is the input to the model. **A. Schematic overview of the metabolic feedback model structure.** oHb and dHb are oxyhemoglobin and deoxyhemoglobin, respectively. **B. Schematic overview of the neurotransmitter feed-forward model structure.** The neurotransmitter feed-forward hypothesis is described in more detail in [16]. Green area = astrocyte, blue area = neuron, grey area = blood vessel. Calcium neuron and calcium astrocyte = calcium ion (Ca^2+^) level in the cell, NO = nitric oxide, cGMP = cyclic guanosine monophosphate, AA = arachidonic acid, EET = epoxyeicosatrienoic acids, PG = prostaglandins and 20—HETE = hydroxyeicosatetraeonic acid. Pointed arrows signify positive interactions and flat arrows signify inhibition. Specific implementations and interaction graphs are available in S1 Appendix.

The metabolic model is centered around a feedback control loop, where the rate of blood flow is altered to keep the blood glucose or oxygen levels constant. A schematic overview of this model is shown in Fig 2A. A specific implementation of this hypothesis, *M*_*m*3_, is plotted in Fig B in S1 Appendix. Fig B in S1 Appendix is an *interaction graph*, which means that it directly visualizes the interactions included in the model structure, as described in *e.g.* [23][24]. More specifically, this means that the non-regulated rates are given by mass-action kinetics, and each differential equation is given by the sum of the in- and out-going reactions, weighted by the stoichiometric matrix. There are three exceptions to this interpretation. First, the blood flow is a variable, not a state, and it affects all four states oxyhemoglobin (oHb), dHb, glucose, and molecular oxygen (O_2_) by transporting the species in and out of the studied vessel. Second, the stoichiometries of the metabolism in the stimulated and the basal states are different and not specified in Fig B in S1 Appendix. Finally, the delay boxes means that intermediate states have been introduced for *e.g.* glucose and its influence on the blood flow. This means that the ODE for *e.g.* oHb is given by

$$\frac{d[\text{oHb}]}{dt}=k_{1f}\left[\text{dHb}\right]\left[O_{2}\right]-k_{1b}\left[\text{oHb}\right]+v_{\text{flow}}{\left[\text{oHb}\right]}_{\text{basal}}-v_{\text{flow}}\left[oHb\right]$$ (5)

where *k*_1f_ and *k*_1b_ are reaction rate constants; where *v*_flow_ denotes the blood flow, and where [oHb]_basal_ denotes the concentration of oHb in the blood flowing in to the studied area. Similar equations describe states and reactions seen in Fig 2A. All equations and model parameters are specified in detail in S1 Appendix, where also all scripts used for the analyses in the paper can be found. The underlying assumptions of the model are discussed in Section “Assumptions and Limitations”.

The neurotransmitter hypothesis is centered around a feed-forward signaling system, where the regulation of the blood flow is given by the balance of positive and negative regulations. The first model structure corresponding to this hypothesis, *M*_*n*1_, is depicted in Fig 2B. The ODEs are directly specified by the interaction graph in Fig C in S1 Appendix, using standard and already mentioned conventions. As can be seen in both figures, the input signal triggers release of glutamate into the synaptic cleft. Glutamate increase triggers calcium-permeable non-methyl D aspartate (NMDA) activated channels in neurons and astrocytes to open, letting calcium flow into the cells. In neurons, the calcium influx leads to an increase in the concentration of nitric oxide (NO) that stimulates the production of vasodilating cyclic guanosine monophosphate (cGMP). In the astrocyte, calcium ions increase the production of arachidonic acid (AA), whose metabolites epoxyeicosatrienoic acids (EET), prostaglandins (PG) and hydroxyeicosatetraeonic acid (20-HETE) effect blood vessel radii. The interaction graph in Fig C in S1 Appendix is based on a similar figure in Attwell *et al.* [16].

#### Fitting to data

Once the model structure has been formulated (Fig 1C, Step 1 in the modeling workflow) and data has been collected (Step 2, data acquisition described below), the parameters, *p*, need to be determined. The parameter evaluations are centered on the following cost function

$$\chi^{2}\left(p\right)=\sum_{i=1}^{N}\frac{(y\left(t_{i}\right)-\hat{y}{\left(t_{i}|p)\right)}^{2}}{\sigma {\left(t_{i}\right)}^{2}}\in \chi^{2}\left(d\right)+\text{additional terms}$$ (6)

where *y*(*t*) are the measured data points at time *t*; where $\hat{y}\left(p\right)$ are the corresponding simulated data points at time *t*; where *N* is the number of time-points; and where *σ*(*t*) is the measurement uncertainty at time *t*. The summation in Eq (6) sums the squared and normalized residuals, indicating how far the simulations $\hat{y}$ are from the data *y*. The additional terms (also referred to as “punishments”, “weights” or “penalties”) are included only when additional requirements are needed. Such requirement might be the presence of an initial dip in the BOLD response (as described in the evaluation criteria in Section “Model evaluation”). If the additional requirements are fulfilled, the additional terms equal zero. If the additional requirements are not fulfilled, the additional terms are increased to force the optimization away from such parameter sets.

In practice, the parameters are determined by optimizing *χ*^2^(*p*) over *p*, using the function “simannealingSBAO” in the Systems Biology toolbox for Matlab [25]. In other words, the optimal parameters $\hat{p}$ are given by

$$\hat{p}=\arg\min\chi^{2}\left(p\right)$$ (7)

#### Model evaluation

The final part of the hypothesis testing in the model construction work flow is to check whether the obtained model fulfills the set criteria of the study. In this study the model structure must be able to:

1. Display a statistically acceptable agreement with experimental BOLD response data.
2. Display an initial dip, a peak, and a post-peak undershoot in the simulated BOLD response.
3. Fulfill both of the above criteria in a biologically plausible manner.

The first criterion is tested using the *χ*^2^ test. This test is based on the observation that the cost function in Eq (6) follows a *χ*^2^ distribution if the measurement noise follows a normal distribution, with standard deviation *σ*. Therefore, the resulting cost is compared to the inverse of a cumulative *χ*^2^ distribution, where the degrees of freedom are given by the number of data points minus 1. In practice, one decides whether the cost is acceptable or not by comparing the cost with the cut-off value [18]. In our case this means that the cut-off have been 49.8 (*χ*^2^, *α* = 0.05, df = 35) for the estimation data. The second criterion is evaluated via simple simulations, and their fulfillment is ensured by adding these criteria as additional terms in Eq (6).

It is necessary that the model output can fit the data (Criterion 1), but this criterion is not enough to test the underlying mechanism. Therefore, the second and third criteria are added. Most notably, the initial dip is often absent in collected data, although it has been proposed to carry important information about the underlying neuronal activity (discussed further in Discussion: The Initial Dip). The second criterion ensures that the model has the mechanisms required to simulate an initial dip if it is fitted to a data set where it is expressed.

The third criterion is more vaguely phrased, as biological plausibility depends on the mechanisms present in each specific model structure. The third criterion is therefore developed and tested separately for each model structure.

#### Model minimization and comparison

Mechanistic models describing biological systems often become complex with several interacting states and parameters. Such a model, although good for illustrating the biological hypothesis, is computationally heavy and poses difficulties during overview and analysis. A strategy to analyze how the model describes the main mechanisms behind the BOLD response is model minimization (Step 4 in Fig 1C). Model minimization is done by excluding parts of the original model and optimizing it anew to the data. If the reduced model is still able to describe the data, yet another state or a group of states can be excluded. The minimal model has been reached when no more states or reactions can be excluded without the model loosing the ability to fit to the data.

Reducing the number of parameters in the model often makes it harder for the model to fit the data, and thereby the cost of the model will increase compared to the original version of the model. In order to see if the model fit is good enough to compensate for the increased cost, a likelihood ratio test can be performed. If

$$\chi^{2}\left(n_{m1}-n_{m2}\right)\leq 2*\left(V_{m1}\left({\hat{p}}_{m1}\right)-V_{m2}\left({\hat{p}}_{m2}\right)\right)$$ (8)

then the minimized model is not significantly worse at describing the data than the original model, despite its reduced number of parameters. The symbol *n*_*mi*_ is the number of parameters in the model structure *i* and $V_{mi}\left({\hat{p}}_{mi}\right)$ is the lowest cost that the optimization has found for the same model structure.

#### Core prediction analysis

The final model analysis is referred to as core prediction analysis, seen in Step 5 in Fig 1C. Core prediction analysis is making predictions with uncertainties [21] and [26], which are then tested towards validation data (Step 6 in Fig 1C). More precisely, the predictions are analyzed for not only one, but for all parameter sets that pass a *χ*^2^ test and thereby can describe the estimation data. In practice, we do not analyze the complete set of acceptable parameters. Instead, we create an approximation of this set, where only a limited number of parameters are studied. This limited set is obtained by saving those acceptable parameters that are encountered during the original optimization and during the subsequent analysis. This corresponds to the hypothesis testing in Fig 1, and to Step 1 in [21].

### Experimental data

#### Subjects

Time series data of the BOLD response were collected from the visual cortex of 13 healthy subjects. The subjects were instructed not to consume caffeine, alcohol, or use nicotine on the day of examination. All subjects gave their written informed consent and the study was approved by the Regional Ethical Review Board in Linköping (M74-06).

Individuals failing to fill the criteria set in the standard MR safety screening form were excluded from participating in the study. Maximum allowed translational head movement was limited by a cut-off value of 3.5 mm (the one-dimensional size of one voxel). After reviewing the head movement of each subject, one subject (out of 13) was excluded from further analysis. Thus, 12 subjects (mean age = 23.5 years SD: 4.4, range = 19–35 years) remained in the study. Seven subjects were men and five were women.

#### Visual stimulation

**Fig 3:**
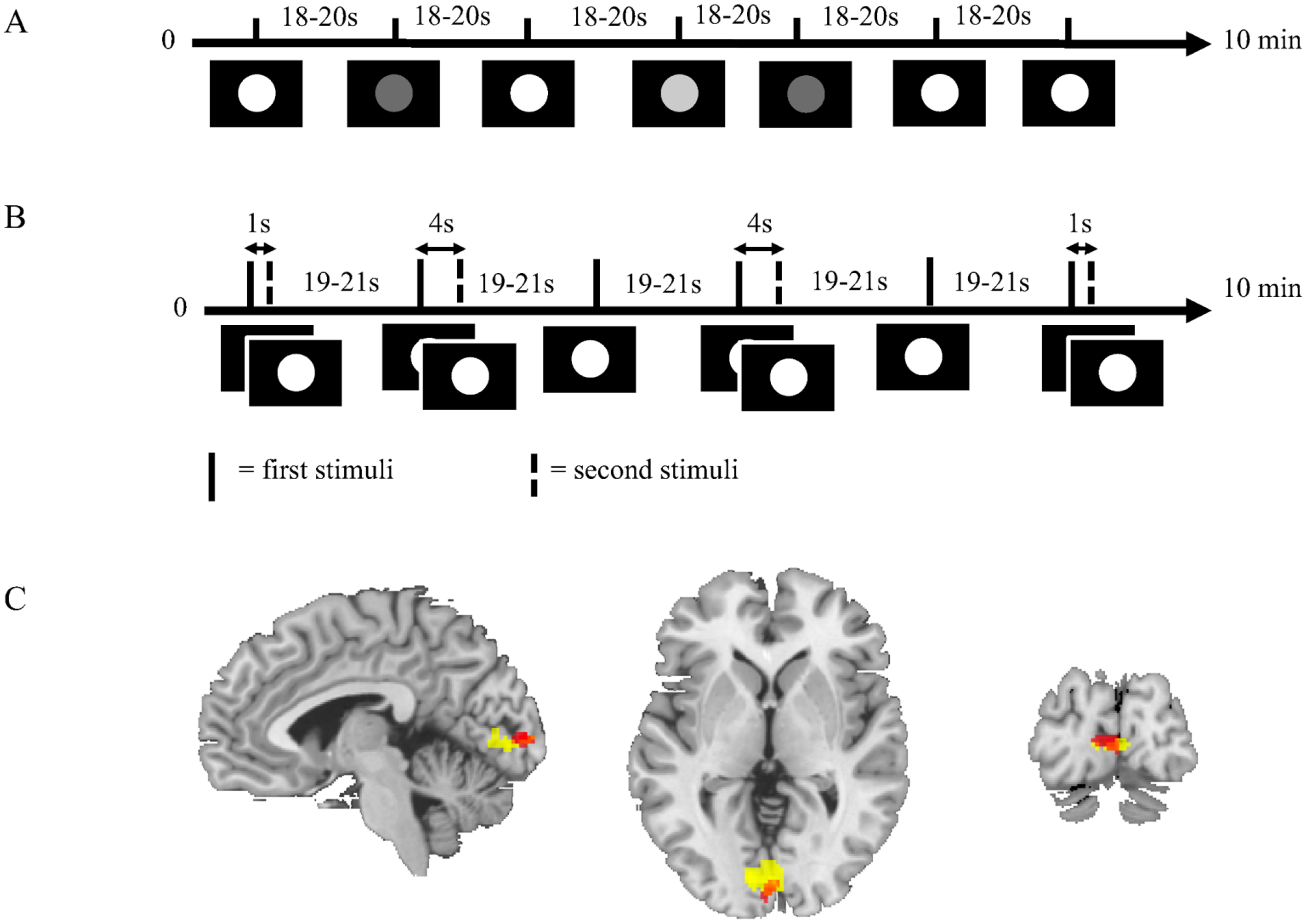
**A. Intensity experimental paradigm.** The figure shows the principal experimental design where the stimuli consisted of circles in white and two shades of grey on a black background. White circle stimulus was the primary stimulus and was used to generate the estimation data. **B. Frequency experimental paradigm.** The figure shows the principal experimental design where the stimuli consisted of white circles on black background. Sometimes a single stimulus was shown and sometimes paired stimuli were shown with inter-pair interval (IPI) of 1 s or 4 s. **C. Group level activation in the visual cortex.** The figure shows activation in the visual cortex during the intensity (red) and the frequency (yellow) experiments across the whole group of subjects. Only activation that passes p = 0.05, family wise error (FWE) corrected threshold is shown in the figure.

The study was designed in the form of two experiments, the *intensity* and the *frequency* experiment (Fig 3A and 3B, respectively). These experiments were based on brief visual stimulation using a sparse event-related design in order to isolate individual BOLD responses. The principal visual stimulus consisted of filled white or grey circles shown for 500 ms on a black background. Each stimulus was followed by a randomly jittered intertrial interval (ITI) to reduce adaptation effects. During the ITI a grey focus cross was presented against black background.

Nine subjects performed the intensity experiment first and the frequency experiment last. Four subjects performed the experiments in the opposite order. In both experiments the visual stimuli were presented using video goggles (VisuaStimDigital, Resonance Technology Inc., USA) with a built in screen and correction lenses. The experimental paradigm was presented using the software package SuperLab 4.5 (Cedrus Corporation, San Pedro, CA, USA) using a Windows XP computer. The presentation of the stimuli was randomized using SuperLab’s randomize function, randomizing once per group of participants. Thus, the stimulus onset times did not vary between individuals.

The intensity experiment and the frequency experiment had three stimuli each. In both experiments, the single, bright white circle shown for 500 ms, was used as primary stimulus. The time course of the primary stimulus of each experiment was used as estimation data when the models were fitted.

In the intensity experiment (Fig 3A), the color of the circle was white, light grey, or dark grey. Each stimulus was presented a total of 9 times each with an ITI of 18 to 20 seconds. Thus, the experiment contained 27 trials with a total runtime of approximately 10 minutes.

The frequency experiment (Fig 3B) included the bright white circle on black background presented in three different frequency modes. The first mode consisted of the primary stimulus, which was the same as in the intensity experiment. In the other modes, the stimuli were paired, with an inter-pair interval (IPI) of 1 or 4 seconds, respectively. Each stimulus was presented 8 times each with an ITI of 19 to 21 seconds. The experiment contained 24 trials with a total runtime of approximately 10 minutes.

#### MRI

All experiments were performed with a Philips Ingenia 3 T MR scanner and a 24-channel head coil. BOLD-images were acquired using a gradient echo sequence sensitive for the BOLD contrast using the following parameters: repetition time (TR) = 500 ms, echo time (TE) = 30 ms, resolution = 3.5 mm isotropic, field of view = 224 mm × 196 mm × 35 mm, flip angle = 60 degrees, echo planar imaging (EPI) factor = 29, sense factor = 2.2. Ten axial slices were collected, oriented from the calcarine sulcus to the cingulate gyrus. The number of acquired volumes (number of dynamics) was 1160 per experiment. T_1_-weighted (T1W) scans were obtained for each individual, as a basis for co-registration of the BOLD-images to high-resolution anatomical images. The following parameters were used for T1W imaging: field of view = 240 mm × 240 mm × 180 mm, voxel size = 0.5 mm × 0.5mm × 0.6 mm, TR = 13 ms, and TE = 6.3 ms.

#### Image analysis

Images from each subject were preprocessed using SPM8 (www.fil.ion.ucl.ac.uk/spm). All images were re-aligned to the first image in the time series to correct for motion during scanning. Thereafter the images were co-registered to the T1W anatomical reference and normalized to the standard template in MNI (Montreal Neurological Institute) space. The normalized images were smoothed with 7 mm Gaussian kernel to reduce noise and ameliorate inter-subject differences in brain anatomy during the voxel wise group analyses.

BOLD-images from all individual subjects were analyzed using the canonical hemodynamic response function implemented in SPM8. A one-sample t-test was used to identify the peak activation in the visual cortex of the study group. This result was used to guide the extraction of the BOLD responses in individual subjects. The BOLD responses were extracted from the un-smoothed images of each individual in native space using a sphere with radius 5 mm around the individual subject’s peak activation in the visual cortex. The individual peak activation was conjointly estimated for both experiments and all stimulations. MarsBaR [27] was used to create the spherical masks. BOLD time series data were normalized to baseline by subtracting the signal value of the last time point before the stimulation from the values of the entire BOLD time series corresponding to each stimulus. BOLD responses from each stimulus were first averaged over each individual and thereafter over the group.

## Results

### Brain activation during visual stimulation

Visual stimulation during both the intensity and the frequency experiment elicited significant activation in bilateral primary visual cortex, p < 0.05 family wise error (FWE) corrected for multiple comparisons (Fig 3C). FWE is a Bonferroni-correction applying the random-field theory (RFT) to control the FWE rate by assuming that the data follow certain specified spatial patterns [28]. The Montreal Neurological Institute (MNI) co-ordinates of the activation peaks were: [-2, -96, 4] and [-10, -84, 2] for the intensity and the frequency experiments, respectively.

**Fig 4:**
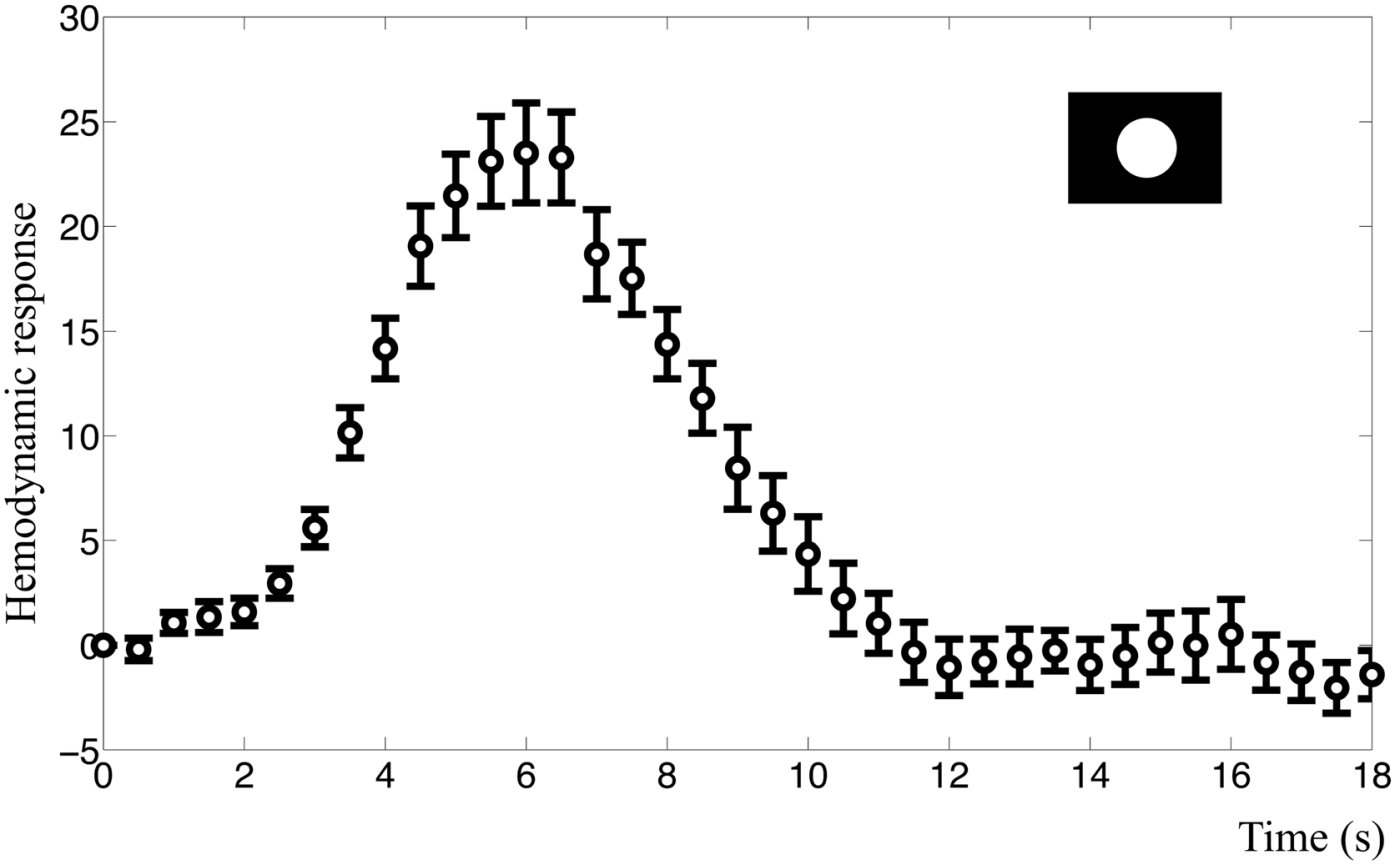
BOLD response in the visual cortex. The figure shows the mean BOLD response and standard error for the primary visual stimulus of the intensity experiment. This time course was used as estimation data when the models were fitted. The *y*-axis represents normalized MR signal in arbitrary units.

The mean BOLD response to the primary visual stimuli in both experiments had a characteristic response peak at approximately 6 s after the stimuli (Fig 4). Peak amplitude was 23.5 (2.12% signal change) in the intensity experiment and 19.8 (1.95% signal change) in the frequency experiment. We also observed a post-peak undershoot, but neither of the resulting BOLD responses in any subject displayed a clear initial dip.

### Rejection of the metabolic feedback model structure

#### Blood flow needs to be controlled by glucose

When implementing the metabolic feedback model initial attempts were made with a model structure, *M*_*m*1_, where oxygen levels controlled the blood flow, but such a model structure could neither fulfill the criteria of displaying a post-peak undershoot nor fit the data, as can be seen in Fig A in S1 Appendix. The model structure was therefore rejected at Steps 2 and 3 in the modeling workflow (Fig 1C). If glucose instead of oxygen was selected to control blood flow, it was possible to simulate the shape of the BOLD response by varying the degree of aerobic *vs.* anaerobic metabolism, as is done in model structure *M*_*m*2_.

#### Stimulated metabolism must be partly anaerobic to obtain both an initial dip and a peak

**Fig 5:**
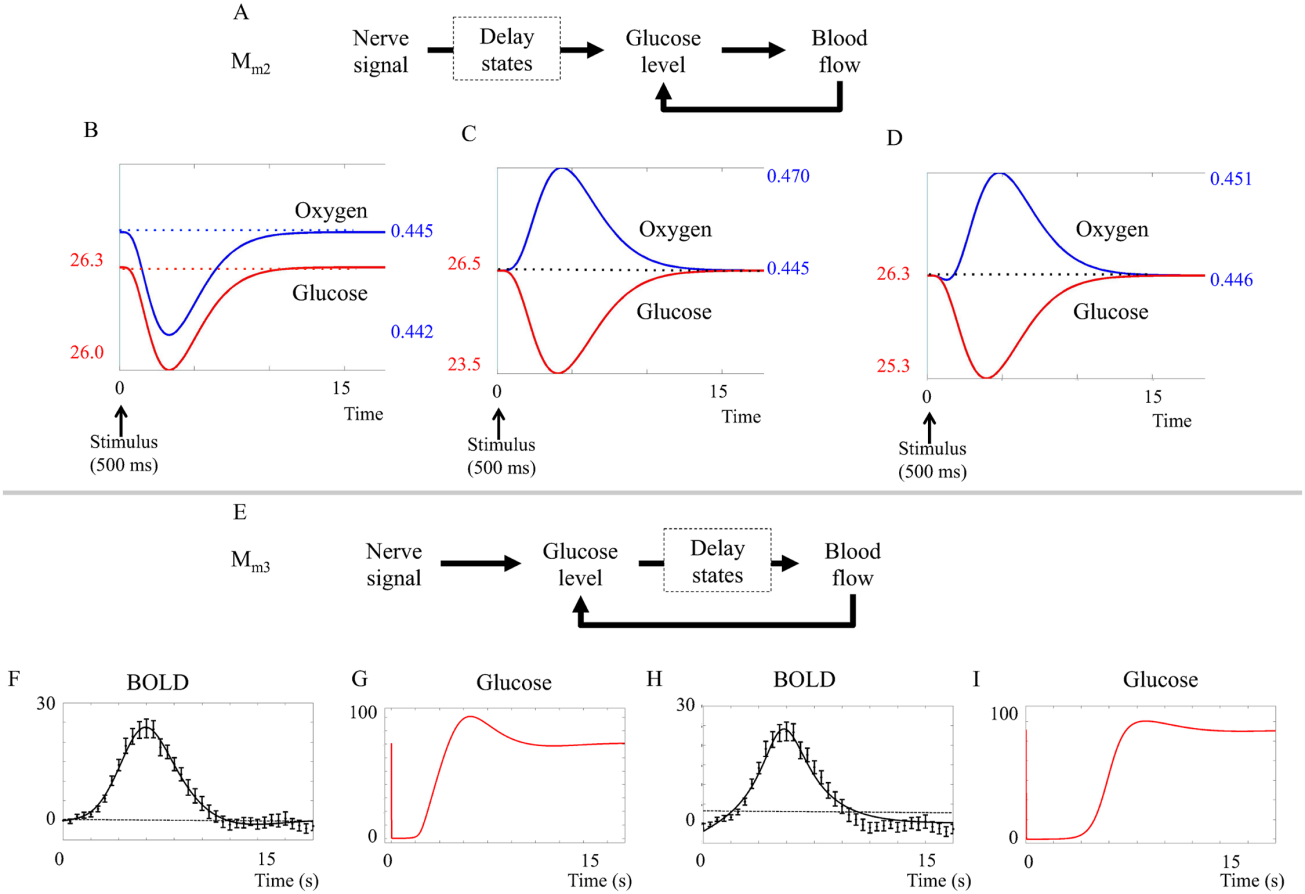
Results from the metabolic feedback model. **A. Placement of the delay states (dashed squares) in model *M*_*m*2_.** The delay states are placed mainly between the neuronal activity and the glucose metabolism. **B-D. Impact of aerobic versus anaerobic metabolism.** B. Predicted BOLD response of *M*_*m*2_ assuming (*CMRO*_2_/*CMR*_*glu*_)_*basal*_ = (*CMRO*_2_/*CMR*_*glu*_)_*stimuli*_. C. Predicted BOLD response of *M*_*m*2_ assuming (*CMRO*_2_/*CMR*_*glu*_)_*basal*_ > (*CMRO*_2_/*CMR*_*glu*_)_*stimuli*_ = 0. D. Predicted BOLD response of *M*_*m*2_ assuming (*CMRO*_2_/*CMR*_*glu*_)_*basal*_ > (*CMRO*_2_/*CMR*_*glu*_)_*stimuli*_ > 0. To display both an initial dip and a peak, the model must have a metabolism that is more anaerobic during stimulation, compared to the metabolism during the basal state. **E. Placement of the delay states in model *M*_*m*3_.** The delay states are placed mainly between the glucose metabolism and the blood flow. **F-G. The glucose response in *M*_*m*3_.** G and H. The best fit does pass a Chi-square goodness-of-fit test, but lacks an initial dip and glucose goes down to zero for several seconds. H-I. *M*_*m*3_ can be forced to display an initial dip, but then it has no undershoot and glucose must still go down to almost zero in order for the feedback to kick in.

Further tests of *M*_*m*2_ in the hypothesis testing revealed an important insight regarding a necessary difference between the basal metabolism and the stimulated metabolism. This difference concerns the relationship between oxygen and glucose consumption. If the metabolism is continuously aerobic, *i.e.* if the ratio of oxygen and glucose consumption is equal during basal state and stimulation, the effect of increased metabolism is oxygen level reduction ($M_{m2}\left({\hat{p}}_{1}\right)$, Fig 5B, first three seconds). This oxygen reduction persists until the blood flow is sufficiently up-regulated to normalize oxygen levels (at approximately 10 seconds, Fig 5B). In other words, in this situation, there is an initial dip but no peak.

Conversely, if the stimulated metabolism is completely anaerobic, *i.e.* if glucose but not oxygen metabolism is increased during stimulation, the effect would be an increase in blood flow to compensate for decreased glucose levels. Oxygen levels will then increase as the increased blood flow brings more oxygen to the capillaries, but this extra oxygen is not metabolized. In this situation (which can be seen in $M_{m2}\left({\hat{p}}_{2}\right)$, simulated in Fig 5C) there is a peak but no initial dip.

A combination, $M_{m2}\left({\hat{p}}_{3}\right)$, where the stimulated metabolism is adjusted to be more anaerobic than the basal metabolism, both an initial dip and a following peak is produced (Fig 5D). In practice, the proportion of aerobic and anaerobic metabolism was varied by changing the number of oxygen molecules used during glucose metabolism. The parameter sets where the proportion parameters are changed can be seen in section 1.2.3 in S1 Appendix. We did not find any versions of the model that did not require a more anaerobic metabolism during stimulation, and we therefore conclude that, if the metabolic feedback hypothesis is true, the metabolism must be more anaerobic during stimulation compared to the metabolism during basal state.

#### The metabolic feedback model structures *M*_*m*2_ and *M*_*m*3_ have problems with predicted glucose levels

The metabolic feedback model structure *M*_*m*2_ assumes glucose regulation of blood flow and a more anaerobic process during stimulation. *M*_*m*2_ has a statistically acceptable fit to the intensity data primary stimulus according to *χ*^2^ goodness-of-fit test (cost = 45.5, cut-off = 49.8). However, as can be seen in Fig 5D, the glucose state decreases very slowly. This means that glucose minimum occurs simultaneously with the BOLD response peak, *i.e.* simultaneously with the oxygen peak. This behavior contradicts the basic principle of the metabolic hypothesis and is caused by delay states inserted between the neuronal signal and the glucose metabolism in the model structure (dashed square in Fig 5A). These delay states between the stimulus and the glucose metabolism contribute to the shape of the initial dip, but have no biological interpretation.

A new model structure, *M*_*m*3_, was constructed. In the model structure *M*_*m*3_ (Fig 5F), the delay states are placed between the metabolism and the blood flow, and represent the action of smooth muscle controlling the radius of the blood vessels. The fit of ($M_{m3}\left({\hat{p}}_{4}\right)$) and ($M_{m3}\left({\hat{p}}_{5}\right)$) is shown in Fig 5F and 5G. As can be seen, the model structure *M*_*m*3_ is incapable of displaying both the initial dip and a post-peak undershoot simultaneously. Furthermore, both fits of *M*_*m*3_ entail problems with the predicted glucose levels. The graphs depicting glucose levels (Fig 5G and 5I) show that the glucose levels decrease to almost zero (< 5% of the original value) within the first 2 seconds after the stimulus, and in the case of an initial dip (Fig 5I), remain low until the peak of the BOLD response has passed. Even though the exact stimulated glucose dynamics is unknown, we consider this predicted behavior unrealistic.

#### The metabolic feedback hypothesis *M*_*m*_ is rejected

In summary, the metabolic feedback hypothesis *M*_*m*_ can describe the experimental data, but is still rejected during the hypothesis testing for two reasons. Firstly, it cannot produce both an initial dip and a post-peak undershoot in the same simulation. Secondly, and most importantly, the tested metabolic feedback model structures are not biologically plausible, because they predict depletion of glucose levels and unrealistically fast and/or slow time course of stimulated glucose dynamics.

### Rejection of the neurotransmitter feed-forward model structure

#### Fitting of the neurotransmitter feed-forward model

**Fig 6:**
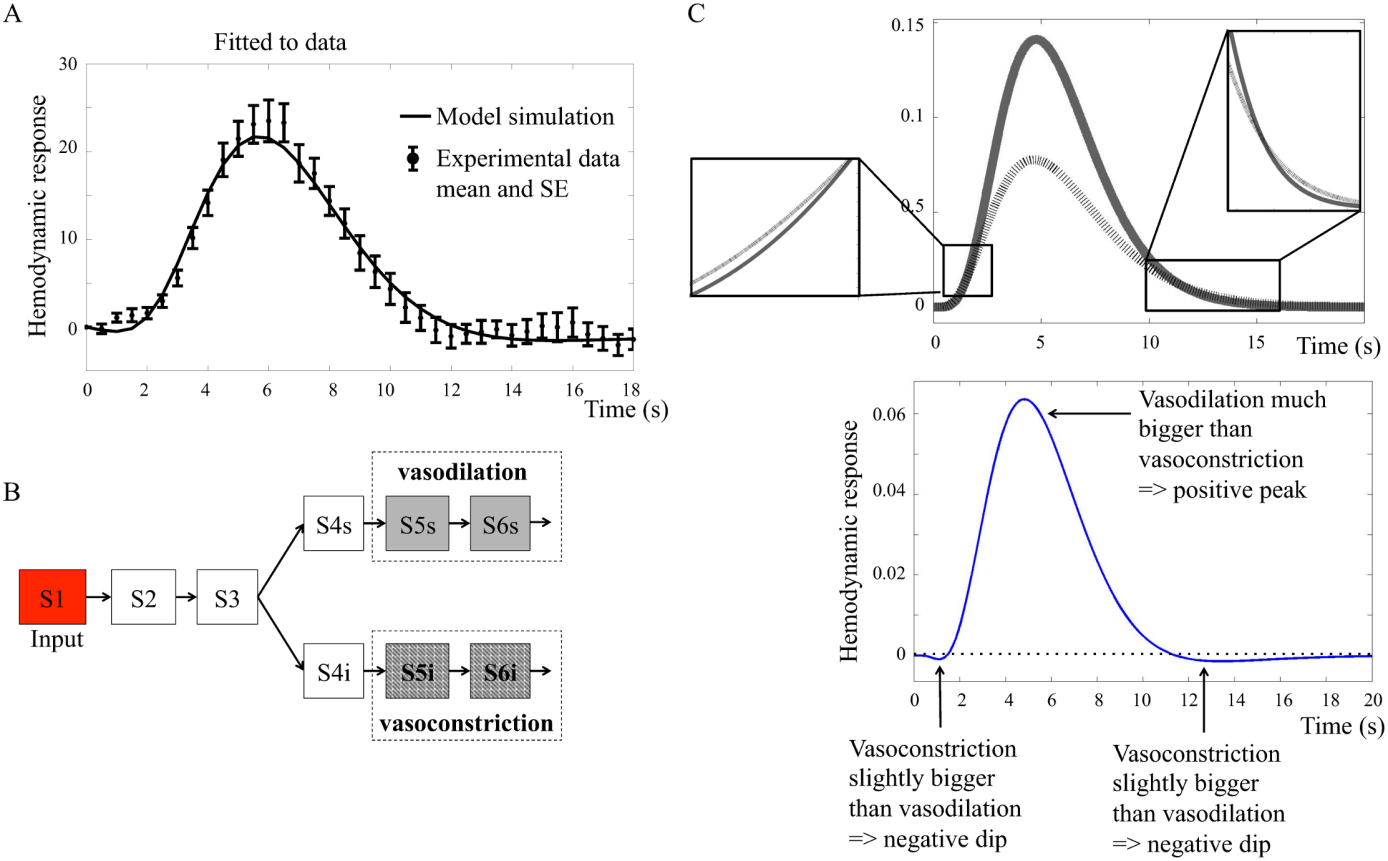
**A. Neurotransmitter feed-forward model $M_{n1}\left({\hat{p}}_{6}\right)$ fitted to data.** The figure shows mean values and standard error (SE) from primary stimulus in the intensity experiment. **B. Interaction graph of the minimized model *M*_*n*2_.** Vasodilating (grey) states contribute positively to the output term and vasoconstricting (hatched) states contribute negatively. S1 … S6 are different states in the model. The behavior of these states are represented with the same color coding in C. **C. (upper panel) Main mechanism of *M*_*n*2_.** The solid grey line shows the action of the vasodilating terms and the black hatched line shows the behavior of the vasoconstricting terms. The insets show the small, but essential, differences that give rise to the initial dip and the post-peak undershoot. **C. (lower panel) Resulting BOLD response from *M*_*n*2_.** The vasodilating states minus the vasoconstricting states give rise to a BOLD response with initial dip, peak, and post-peak undershoot.

The neurotransmitter feed-forward model $M_{n1}\left({\hat{p}}_{6}\right)$ can fit the estimation data from the primary stimulus. As can be seen in Fig 6A, the neurotransmitter feed-forward model clearly displays the typical peak in the BOLD response and the characteristic initial dip and post-peak undershoot. Since the hypothesis presented in Attwell *et al.* [16] focuses on the neurovascular control of the blood vessels, there is no metabolism, and under this assumption, the blood flow can be used as a direct proxy for the output, i.e. for the oxygen, dHb, and oHb levels as the output of the model structure *M*_*n*1_.

#### The main mechanism in the neurotransmitter feed-forward model is a balance between vasoconstriction and vasodilation

The neurotransmitter feed-forward model structure *M*_*n*1_ is complex with several states, which includes most of the signaling molecules mentioned in the review by Atwell *et al.* [16]. In order to investigate the key mechanisms of the neurotransmitter feed-forward hypothesis, *M*_*n*1_ was minimized. Fig 6B shows the minimized neurotransmitter feed-forward model $M_{n2}\left({\hat{p}}_{2}\right)$ that consists of the simplest combination of states which can still describe the typical BOLD response (fit to data shown in Fig E in S1 Appendix). *M*_*n*2_ shows that the main mechanism in the neurotransmitter feed-forward model is described by one vasoconstricting and one vasodilating process, shown by the hatched and filled lines respectively in Fig 6C. In order to model a BOLD response with initial dip, peak and post-peak undershoot, it is necessary to have a constricting output term that rises early, but to a lower amplitude than the dilating output term, and falls slowly back to baseline. The dilating output term has a later but quicker rise to high amplitudes, and falls more quickly back to baseline compared to the dilating term.

#### The neurotransmitter feed-forward model predicts vasoconstriction to cause the initial dip

The neurotransmitter feed-forward model $M_{n1}\left({\hat{p}}_{6}\right)$ can display all characteristic features of the BOLD response *i.e.,* the initial dip, the peak, and the post-peak undershoot. $M_{n1}\left({\hat{p}}_{6}\right)$ can also fit the estimation data. However, when investigating the biological mechanisms, we observed that the initial dip, according to the model, is caused by a constricting output term that rises earlier than the dilating term. That is to say, the neurotransmitter feed-forward model predicts vasoconstriction to cause the initial dip. As previous research suggests that the initial dip most probably is related to oxygen metabolism [3], we reject the neurotransmitter feed-forward model. However, as both the existence of and the mechanisms behind the initial dip is debated, this issue is further addressed in the Discussion.

#### The neurotransmitter feed-forward hypothesis is rejected

In summary, the neurotransmitter feed-forward model structure *M*_*n*1_ can describe the experimental data, and it can produce an initial dip, peak, and post-peak undershoot. However, it is rejected during the hypothesis testing, because the hypothesis is not biologically plausible, as the initial dip is caused by vasocontraction instead of oxygen metabolism.

### An extension of the neurotransmitter model structure fulfills the biological plausibility criteria

**Fig 7:**
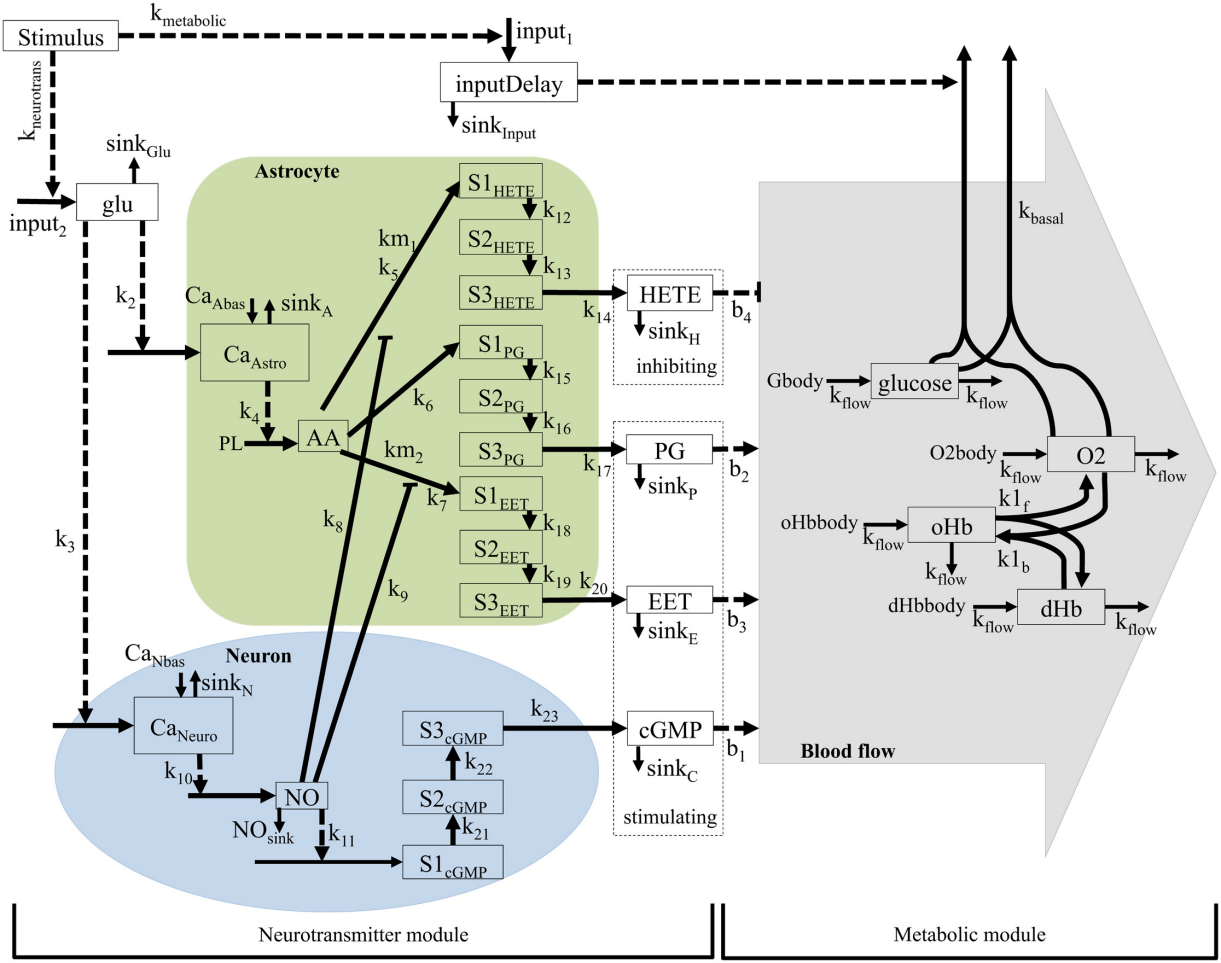
Interaction graph of the extended model structure *M*_*nm*1_. The model structure has two modules: the neurotransmitter module, which controls the blood flow, and the metabolic module, which controls the oxygen and glucose metabolism. Whole squares = states, dashed squares = variables (dependent on states), whole arrows = transformations, dashed arrows = interactions, green area = astrocyte, blue area = neuron, grey area = blood. All states starting with S and a number (e.g S2PG) are delay states. Stimulus = input signal. oHb and dHb are oxyhemoglobin and deoxyhemoglobin, respectively. Glu = glutamate, Calcium neuron and calcium astrocyte = calcium ion (Ca2+) level in the cell, NO = nitric oxide, cGMP = cyclic guanosine monophosphate, AA = arachidonic acid, EET = epoxyeicosatrienoic acids, PG = prostaglandins and 20—HETE = hydroxyeicosatetraeonic acid. All terms starting with k (e.g k1), are parameters and in most cases represent rate constants. PL is a parameter representing phospholipase A2, which is present in abundance. Gbody, O2body, oHbbody and dHbbody, are variables representing the glucose, oxygen and hemoglobin delivered into the area.

Results described above show that the increased oxygen metabolism in the metabolic feedback hypothesis can produce an initial dip and that the neurotransmitter feed-forward hypothesis can give a realistic description of the blood flow increase during the BOLD response. Therefore, the neurotransmitter model structure was extended with a metabolic module, *M*_*nm*1_ (Fig 7). In *M*_*nm*1_, the neuronal activity increases metabolism in the metabolic module and at the same time triggers glutamate release in the neurotransmitter module. The levels of dHb and oHb are controlled by the metabolic module and the blood flow is controlled by the neurotransmitter feed-forward module. *M*_*nm*1_ has no feedback control of the blood flow. The output of this final model structure is the ratio of dHb and oHb.

#### The extended feed-forward model has realistic biological mechanisms

**Fig 8:**
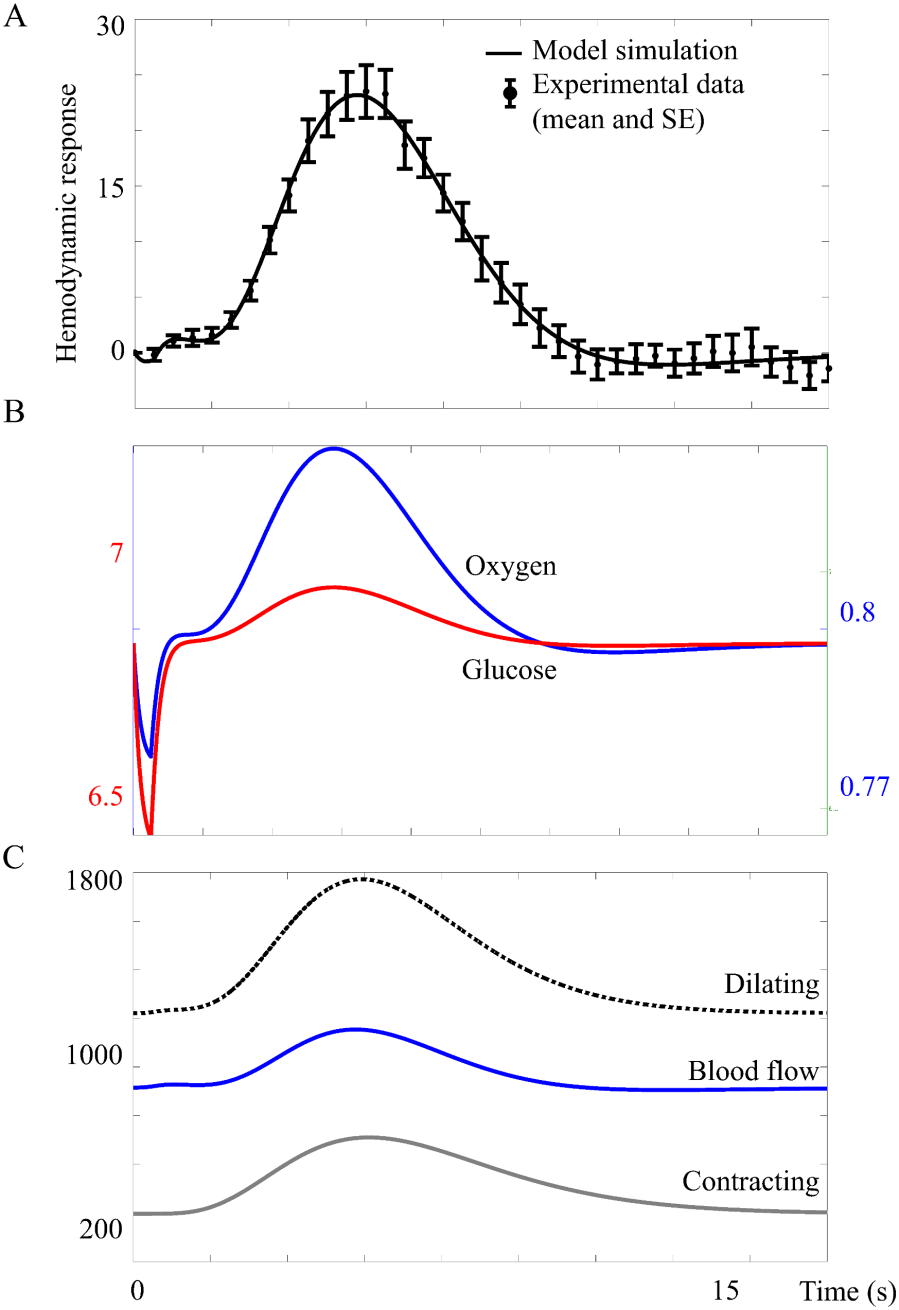
**A. The model structure $M_{nm1}\left({\hat{p}}_{7}\right)$** fitted to data. The figure shows mean values and standard error (SE) from primary stimulus in the intensity experiment. Here the model was forced to display an initial dip and a post-peak undershoot. **B. Glucose and oxygen in $M_{nm1}\left({\hat{p}}_{7}\right)$.** Reduced, but not depleted, glucose level triggers blood flow increase. The increased oxygen metabolism after stimulus causes the initial dip. **C. Vasoconstrictive inhibition of blood flow during the initial dip does no longer occur.**

The model structure *M*_*nm*1_ bridges the gap between cellular action and the regulation of the BOLD response on a vascular level. The model structure *M*_*nm*1_ can fit data from the primary stimuli in both experiments, see Fig 8A. As can be seen in Fig 8B, the final model has an early and moderate glucose metabolism (the glucose level drops about 5% during the first second). In Fig 8B, it can also be noted that oxygen drops during the first second while in Fig 8C the blood flow is stable during the first seconds, indicating that oxygen metabolism causes the initial dip. The blood flow causing the peak and undershoot is controlled by the neurotransmitter feed-forward module (Fig 8C, peak at 6 s).

#### Minimization of the extended neurotransmitter feed-forward model structure

The extended model structure *M*_*nm*1_ represents the system described in Atwell *et al.* [16] and contains the key biological elements described there. However, the model structure of *M*_*nm*1_ can be simplified without loosing the essential mechanisms of the neurovascular coupling. Therefore, *M*_*nm*1_ was minimized to the minimal model structure *M*_*nm*2_ (seen in Fig H in S1 Appendix). The minimization was done by removing the parallell pathways controlling vasodilation and other key biological elements, with the criterion that *M*_*nm*2_ pass both the the likelihood ratio and the *χ*^2^ test. If any more such states were removed, the cost of the model fit increased drastically and the model passed neither the likelihood ratio nor the *χ*^2^ test. The 49 parameters of *M*_*nm*1_ were reduced to 27 in *M*_*nm*2_. The lowest cost of any parameter set found for *M*_*nm*1_ was 11.2 for the primary stimulus of the intensity experiment and 7.2 for the primary stimulus of the frequency experiment. The lowest cost for the minimal model structure *M*_*nm*2_ in the same experiments were 27.5 and 18.8, respectively. Cutoff for the likelihood ratio test was 33.9 (df = 22), and thus the model structure *M*_*nm*2_ passes the likelihood ratio test despite the decreased number of parameters. The minimal model structure *M*_*nm*2_ does not separate between neurons and astrocytes, but retains only the principle of a dilating and a constricting arm controlling the blood flow. Just as in *M*_*nm*1_ before the minimization, increased oxygen metabolism causes the initial dip in *M*_*nm*2_, while the blood flow remains stable during the first seconds after the stimulus.

#### Predictions of the intensity and frequency experiment validation data

**Fig 9:**
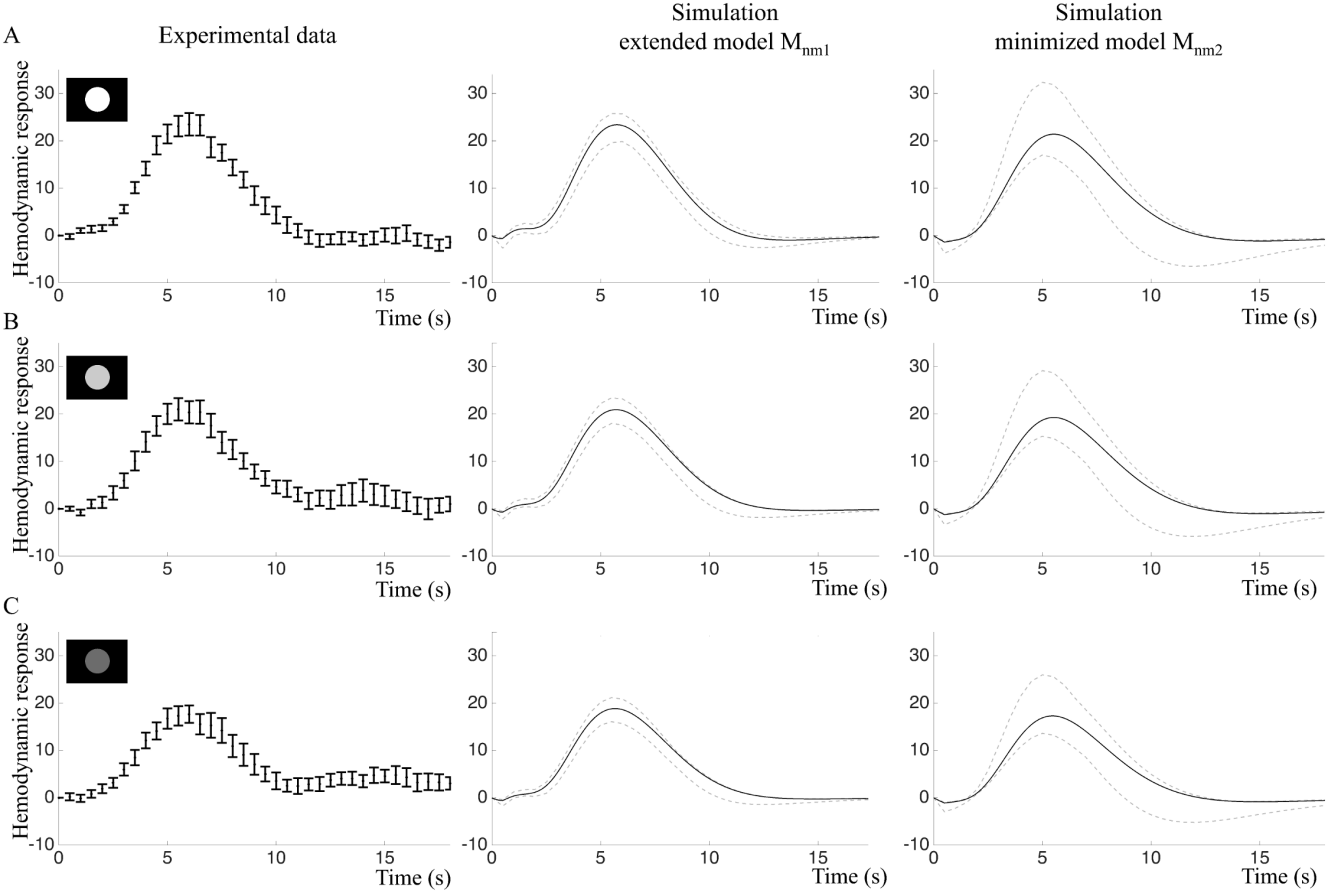
Intensity experiment: Fitting and predictions of the extended model *M*_*nm*1_ and the minimized model *M*_*nm*2_. A. Left: Estimation data (mean and SE) of the primary stimulus in the intensity experiment. Middle and right: Model simulations optimized to the estimation data. Black lines represent the best fit and grey lines represent the maximal or minimal value at that time point from a representative selection of acceptable parameter sets. B. Experimental validation data (left) and core predictions (middle and right) for the light grey stimulus. C. Experimental validation data (left) and core predictions (middle and right) for the dark grey stimulus.

In order to further test the model structures *M*_*nm*_, we made core predictions of the BOLD responses to all stimuli in the intensity and frequency experiments according to the model construction work flow described in Fig 1C, Step 5, and validated the core predictions with the new data (Fig 1C, Step 6). Fig 9 shows that the intensity experiment validates the predictions of both the extended model $M_{nm1}\left({\hat{p}}_{7}\right)$ and the minimized model $M_{nm2}\left({\hat{p}}_{8}\right)$. BOLD responses from the intensity experiment were simulated by changing the amplitude of the input. As the true experimental stimulation intensity for the three types of stimulation (white, light grey and dark grey circles) were not known, only the qualitative behavior of the BOLD response was predicted. Both model structures predict decreased BOLD response peak amplitudes in response to a decreased input signal (Fig 9B and 9C, middle and right panels). Experimental data (Fig 9B and 9C, left panels) confirmed this prediction and indicated that visual stimuli with lower intensity had lower amplitudes of the BOLD response. However, this decrease was not statistically significant for our small sample, *p* = 0.058 in repeated measures ANOVA (Fig 9, right panels). The white stimulus resulted in the highest mean amplitude, 25.5 au (sd = 7.4) and the dark grey stimulus in the lowest mean amplitude, 20.1 au (sd = 8.2).

**Fig 10:**
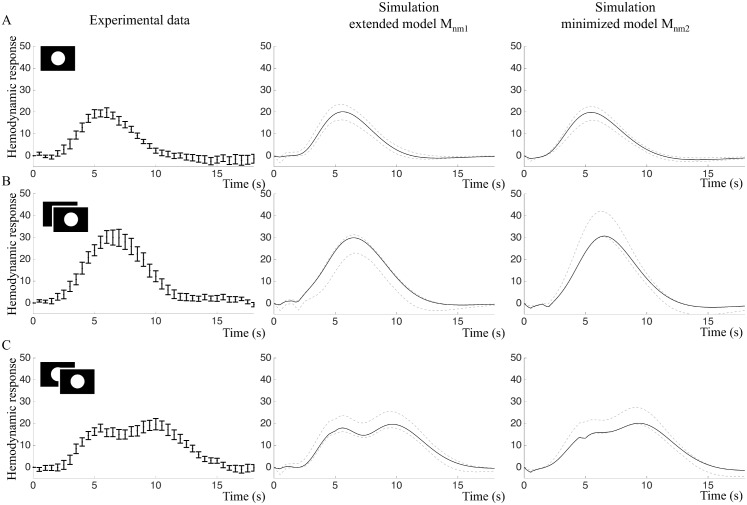
Frequency experiment: Fitting and predictions of the extended model *M*_*nm*1_ and the minimized model *M*_*nm*2_. A. Left: Estimation data (mean and SE) of the primary stimulus in the frequency experiment. Middle and right: Model simulations optimized to the estimation data. Black lines represent the best fit and grey lines represent the maximal or minimal value at that time point from a representative selection of acceptable parameter sets. B. Experimental validation data (left) and core predictions (middle and right) for the 1 s IPI stimulus. C. Experimental validation data (left) and core predictions (middle and right) for the 4 s IPI stimulus.

In the frequency experiment, the amplitude of the input signal is constant but the stimuli are repeated with 1 s or 4 s IPI. Here, quantitative traits of the data were also predicted. To account for changing basal conditions, the model was re-optimized and the time course from the primary stimulus in the frequency experiment (Fig 10A, left panel) was used as estimation data, yielding $M_{nm1}\left({\hat{p}}_{9}\right)$ and $M_{nm2}\left({\hat{p}}_{10}\right)$.

For paired stimuli with 1 s IPI, both models predicted a single BOLD response peak with higher amplitude compared to the single stimulus (Fig 10B, middle and right panel). For paired stimuli with 4 s IPI, the extended model *M*_*nm*1_ predicted a BOLD response doublet with approximately the same amplitude as the single stimulus (Fig 10C, middle panel), while the minimized model *M*_*nm*2_ predicted that the second peak should be higher than the first (Fig 10C, right panel).

The core predictions were then compared to experimental data. The paired stimulus with 1s IPI resulted in a BOLD response with one peak, while the paired stimulus with 4 s IPI produced a BOLD response doublet where the peaks were of approximately equal hight (Fig 10B and 10C, left panels). The 1 s IPI stimulus resulted in a BOLD response with a significantly greater mean amplitude, 33.1 au (sd = 12.8) than the mean amplitude of the single stimulus response, 22.7 au (sd = 7.8), *p* = 0.005 in two-tailed paired t-test. Statistically, both models were able to predict the 4 s IPI data, but as can be seen in Fig 10C, the predictions of the extended model *M*_*nm*1_ has a shape more similar to the validation data compared to *M*_*nm*2_.

## Discussion

We have presented mathematical modeling of the mechanisms underlying the BOLD response in fMRI, based on the metabolic feedback and the neurotransmitter feed-forward hypotheses, extensively discussed in the literature [15][16][29]. These hypotheses describing the fundamental mechanisms behind the BOLD response have, to our knowledge, not been mechanistically modelled before using systems biology approaches. Such approaches provide new tools to evaluate the influence of different hypotheses of the neurovascular coupling causing the BOLD response.

**Fig 11:**
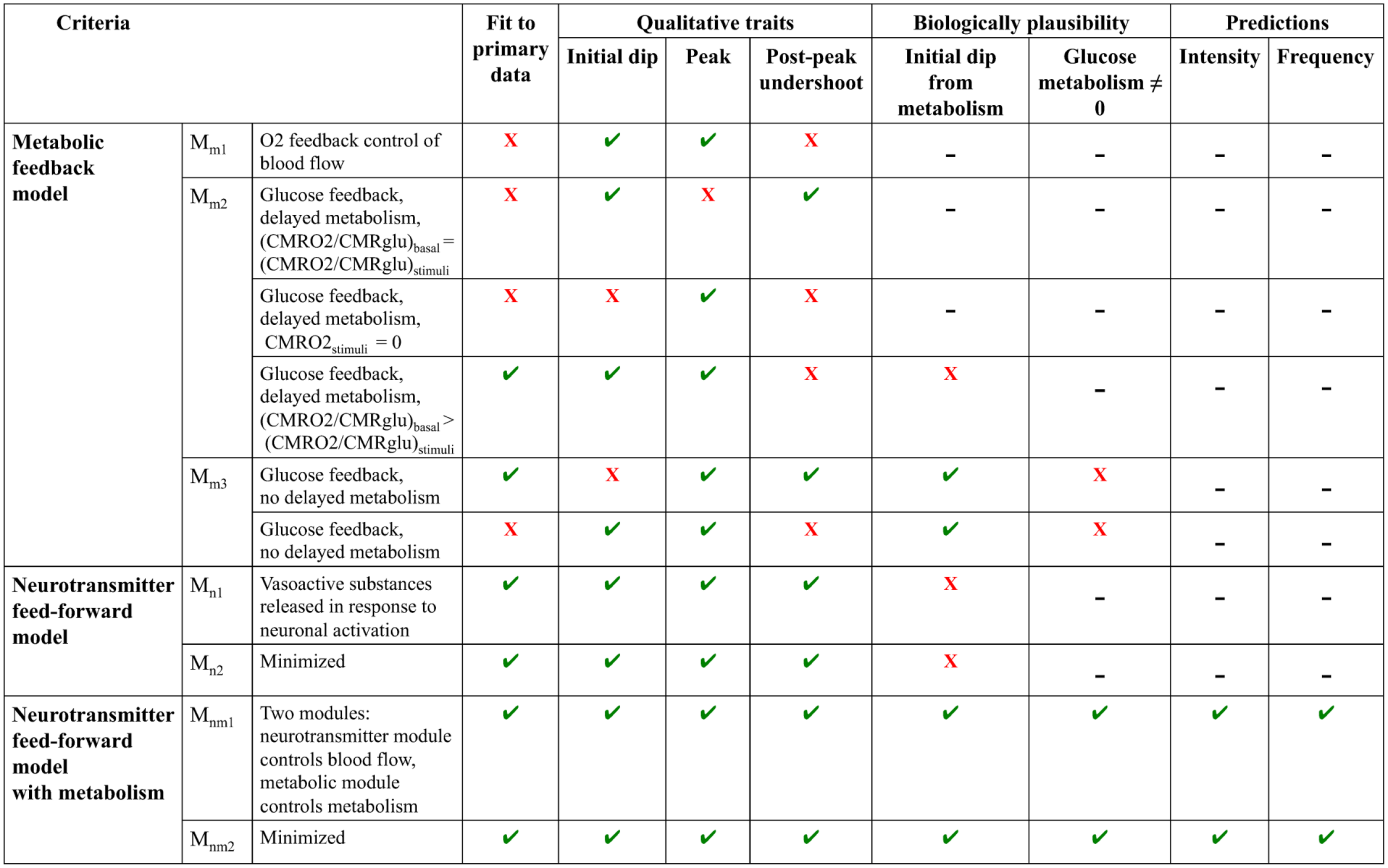
Schematic diagram of rejections and acceptances of the different model structures evaluated in the present study. Only the final models *M*_*nm*1_ and *M*_*nm*2_ fulfill all criteria. ✓ = model passes the test, X = model fails the test, - = test not applicable or not performed because the model failed a previous tests.

The metabolic feedback model structures *M*_*m*1_, *M*_*m*2_, and *M*_*m*3_ have problems fitting the data (Fig 11). The model structures also have problems with simulating the characteristic traits of the BOLD response, (i) the initial dip, (ii) the peak, and (iii) the post-peak undershoot, at the same time. Further, none of the metabolic feedback model structures could describe a biologically plausible time course of the glucose state (Fig 5). The neurotransmitter feed-forward model structure could describe the data and the characteristic traits, but lacked mechanisms for a correct description of oxygen metabolism as the driving force of the initial dip. Of the model structures tested herein, only the model structures *M*_*nm*1_ and *M*_*nm*2_ that combine neurotransmitter control over the blood flow with glucose and oxygen metabolism can fully describe the BOLD response. The model structure *M*_*nm*1_ could also predict the BOLD responses triggered by stimuli in the intensity and frequency experiments that were not present in the estimation data. Based on these results, we argue that an addition of metabolism to the neurotransmitter feed-forward hypothesis explains necessary mechanisms generating the BOLD response.

We will now discuss the biological interpretation and the underlying assumptions of the model structure *M*_*nm*1_.

### Initial dip

The initial dip is stated as a biological criteria that the model must be able to perform in order to be accepted. The existence of the initial dip is debated [4][5]; it has been observed in some studies [30][31], but not in others, at least not in all subjects [30][32]. Several factors can explain the absence of an initial dip in experimental data: (1) The initial dip is reported to be only 1–2% of the baseline signal [30][31]; in other words, given the low signal to noise ratio in fMRI, the shallow dip could easily be undetected. (2) The intersubject variability is considerable in fMRI [33][34], and thus, the subject selection could be decisive for observing an initial dip or not. (3) The observance of an initial dip could be dependent on the experimental design. For instance, Hu *et al.* [30] found that the magnitude of the dip was reduced for brief stimuli; the minimum stimulus duration in their study was 1.5 s, and we used a duration of only 0.5 s, which could possibly explain the absence of the initial dip in our study. Even though the data largely lacked the initial dip, we included the initial dip as a constraint in our models.

The leading hypothesis of the mechanism underlying the initial dip is an uncoupling of the oxygen metabolism from the CBF [4][5], where the increased stimulus-induced oxygen metabolism leads to increased dHb levels and following decreased early-phase BOLD response. This hypothesis has been described previously by a model using the gamma variate curve [13], and is also supported by optical imaging studies showing early stimulus-related dHb increases [35][36]. Based on these previous studies and the simulations of the metabolic feedback model structure in our work, the oxygen metabolism (but not the feedback) of the metabolic feedback hypothesis is necessary, but not sufficient, to reproduce the shape of the BOLD response. However, the metabolic feedback model structure has difficulties explaining the initial dip in combination with a post-peak undershoot (see Fig 5). The neurotransmitter feed-forward model structure, on the other hand, predicts an initial dip caused by initial vasoconstriction, a prediction that is not supported by any previous data (*e.g.* [4][5]) leading to rejection of that model structure. According to our extended model structure, *M*_*nm*1_, the initial dip is caused by changes in dHb/oHb ratio due to increased oxygen metabolism occurring during a CBF delay period, as described above.

### BOLD response peak

The most noticeable feature of the BOLD response is the large stimulus-induced peak of the fMRI signal. Originally it was suggested that reduced blood-oxygen levels increased the CBF [1], and consequently the fMRI signal. This suggestion formed the basis for the model structure *M*_*m*1_. However, we showed that the oxygen-triggered feedback cannot cause such large overshoot (Fig 5A). Later on, Fox *et al.* [15] showed that CBF and CMRO_2_ correlate strongly in the brain at rest, but not in response to stimuli; they found that CBF increased by 50%, CMR_*glu*_ by 51%, but CMRO_2_ only by 5% when the brain is activated. They also concluded that the brain metabolism is aerobic during rest and anaerobic in response to stimuli, to cover the excess energy needed, which is reflected later in the astrocyte-neuron-lactate-shuttle model proposed by Magistretti and Pellerin [37]. Fox *et al.* [15] also showed that regional CBF is not driven by oxidative metabolism, but that stimulus-induced CBF is driven by increased glucose demand (see review by Paulson *et al.* [29]). Prichard *et al.* [38] hypothesized that aerobic glycolysis might be close to its maximum capacity in the resting brain. Therefore, stimulus-induced activity requires quick energy increases via anaerobic glycolysis, causing the uncoupling of glucose and oxygen metabolism and CBF.

In line with previous experimental research [15][29], we show that if metabolism is the driving agent for stimulus-induced CBF then blood flow needs to be controlled by glucose, and stimulated metabolism must be partly anaerobic to obtain both an initial dip and a peak in the BOLD response. However, we also show that the metabolic feedback model structures *M*_*m*2_ and *M*_*m*3_ overstate predicted glucose reduction in response to stimuli, as they predict almost total depletion of glucose to trigger CBF feedback leading to rejection of the metabolic feedback model structure, *M*_*m*_. This suggets that the metabolic feedback hypothesis plays a limited, if any, role in the neurovascular response, a conclusion supported by results from Lindauer et al. [39] and Wolf et al. [40] who showed that an increased CBF response will still occur even when hemoglobin is fully oxygenated and that CBF remains unchanged at hypoglycemia.

The neurotransmitter feed-forward model structure *M*_*n*_ suggests that CBF is regulated in response to neuronal signaling itself and determined by the intensity and duration of the input signal. In our work, *M*_*n*_ predicts all characteristic features of the BOLD response and has acceptable fit to data. By minimizing the model, we could show that the main mechanism in *M*_*n*_ is a balance between vasoconstriction and vasodilation. One caveat with the neurotransmitter feed-forward model is that it predicts vasoconstriction to cause the initial dip, as discussed above.

According to the final model structure *M*_*nm*1_, which has neurotransmitter control of the blood flow combined with metabolism of glucose and oxygen, CBF is primarily controlled by neurotransmitters that initiate processes in neurons and astrocytes causing release of vasoactive agents. The final model also predicts glucose response with similar shape as post-stimulus glucose levels measured by optical methods in rat [41] (Fig 8).

### Post-peak undershoot

The final characteristic of the BOLD response is the post-peak undershoot. According to our final model structure, *M*_*nm*1_, the post-peak undershoot is dependent solely on neurotransmitter-triggered changes in CBF. This result is supported by previous literature that suggests that the post-peak undershoot is caused by a post-stimulus CBF undershoot ([42][43][44] reviewed in [3]). In our data, the post-peak undershoot appears in most of the individual data sets. However, the undershoot seems to become deeper as the peak grows higher in the intensity data sets, and the models tested in the current work cannot predict this behavior. Furthermore, the model cannot describe the deeper undershoot in the 1 s IPI dataset, although it can predict the increased amplitude of the peak.

There are other hypotheses of the mechanisms of the post-peak undershoot that are not investigated here. For example, it is suggested that the post-peak undershoot is caused by slow post-stimulus baseline return of CMRO_2_-related oxygenation or venous CBV [3]. Mandeville and coworkers [45] found slow recovery of CBV that matched the post-peak undershoot duration suggesting a biomechanical rather than a metabolic effect [46]. It is worth noting that the balloon model explains the post-peak undershoot as a slow CBV recovery [6]. It has also been suggested that the post-peak undershoot is modulated by post-stimulus neural activity [47][48]. Future studies incorporating models for CBV changes and/or post-stimulus neural activity might clarify the neurovascular mechanisms behind the post-stimulus undershoot.

### Neurotransmitter and metabolic parameters

The neurotransmitter feed-forward model with metabolism *M*_*nm*1_ contains several experimentally undetermined parameters, such as glutamate and glucose levels. In future studies, the model parameters can be evaluated and optimized using magnetic resonance spectroscopy (MRS) in combination with BOLD-fMRI. In proton MRS, it is possible to obtain time-dependent variations of the neurotransmitters glutamate and GABA and metabolites such as glucose and lactate [49][50][51]. In these recent high-field (7 T) MRS studies, it has been shown that glutamate, GABA, and lactate levels increase during visual stimulation and motor activation, whereas the glucose levels decreases during the same period. If volume was added to the model, a closer estimation of some parameters would also be possible, using experimental values from current literature. This would open the door to prediction of parameter values, in addition to the current predictions of model behaviour.

### Assumptions and limitations

As with all models, the model structures evaluated in this work depend on a number of underlying assumptions, which in turn limit the conclusions that can be drawn from the results. Nevertheless, such assumptions are essential in order to build a comprehensible model. We are well aware that there are several different hypotheses of the mechanisms behind specific features of the BOLD response, of which some are described above. In this work we chose to focus on two fundamental hypotheses.

In the final model structure, *M*_*nm*1_, the metabolic and the neurotransmitter module run in parallel. One of the consequences of this structure is that the metabolism is directly controlled by the input signal (see Fig 7). A more physiologically relevant model would be to integrate the metabolism into neurons and astrocytes. That is to say, to model the glycolysis and oxidative metabolism as actually occurring in the neuronal cells in response to stimuli.

Another part of the model structure that lacks physiological details is the action of smooth muscle cells and effects of cortical vessel elasticity. These mechanisms are in our model represented by delay states, which will not accurately represent the possible non-linearities of receptor actions and muscular contraction or relaxation. In addition, the current model does not differentiate between capillaries and arterioles. Recent research has found that cerebral hemodynamics has a spatiotemporal dependence related to the effective blood viscosity and cortical vessel stiffness [52] and mechanical restrictions on blood vessels depending on cortical depth [53]. A compartmentalized model that takes spatiotemporal hemodynamics into account would provide a physiologically more accurate description of the BOLD response.

Finally, the output signal of the model is oHb/dHb, a simplification suggested by Ogawa *et al.* [1]. However, the BOLD signal equation provides a more correct description of the output signal.

$$\frac{\Delta S}{S_{0}}=e^{-\Delta R_{2}^{*}\cdot TE}-1\approx -\Delta R_{2}^{*}\cdot TE$$ (9)

where *S*_0_ is the MR signal at baseline and Δ*S* is the BOLD signal change with activation. $\Delta R_{2}^{*}$ is the difference in transversal relaxation rate between the activated state and baseline. $\Delta R_{2}^{*}$ is linearly related to dHb concentration. Following ideas from Davis *et al.* [54], several improvements of the BOLD signal description have been published [8][55][56]. In the current work, volumes are not included in the model and therefore it is not possible to implement an output dependent on dHb concentration. However, by dividing the model into a tissue compartment and a blood compartment, as has been done in *e.g.* [13], a more realistic expression for the output BOLD signal can be obtained.

### Balancing complexity and overfitting against ability to predict data

When comparing models with each other, it is important to keep track of model complexity and watch out for potential problems with overfitting. We approach these issues first by choosing a model framework not designed to be as flexible as possible, but instead based on the actual mechanisms believed to be present in the system. Second, we do visual inspection of the plots comparing data and model fits (Figs 9A and 10A). As can be seen in both Fig 9A and 9B, in the time-window 12–18 seconds the mean values in the data show minor fluctuations, which most likely are noise. The model is not following these minor variations, which argues that we do not have problems with overfitting. Nevertheless, in the early time-points (t = 0–3 s), the extended model does an initial dip, stays down a while, and then rises. Since a similar delayed rise can be seen in the data, this could in principle be a sign of overfitting. However, our third approach to test for overfitting—core prediction analysis—argues against that. In the core prediction analysis, we study a representative sub-set of all parameters that describe the data in a statistically acceptable way. In other words, since the core prediction analysis includes both optimal and less optimal parameters, it does not matter if there are some parameters that are overfitted, as long as parameters that are not overfitted are included in the prediction uncertainty analysis. Furthermore, this core prediction analysis shows that all found parameters show an initial dip and a delay (Figs 9A and 10A, middle columns), arguing that this property is a necessary consequence of the model structure and the data, i.e. a uniquely identified core prediction. Our fourth approach for checking for unnecessary over-parametrization is model minimization. This punishes for unnecessary complexity in the sense of parameters that can be removed without significantly worsening the agreement with the estimation data (Figs 9A and 10A, middle and right columns). Finally, the perhaps most important approach to check for overfitting is to use independent validation data. As can be seen in Figs 9B, 9C and 10B, 10C, both the extended model (middle columns) and the minimized model (right columns), agree with this independent data, to which they have not been fitted. Furthermore, as can be seen in e.g. Fig 10C, the original extended model actually agrees slightly better with the data than the minimized model. All these facts argues that our models—although over-parametrized in the sense that many parameters have non-unique values—still are based on realistic biological mechanisms that capture the main features seen in the data, and not on too flexible model structures that are fitting to a specific noise realization.

### Conclusions

Although the BOLD response has been extensively studied and systems biology is a well established method, no one has so far investigated the BOLD response using this type of modeling. In this article, a model based on current physiological hypotheses of the mechanisms behind the BOLD response is presented. The model structures *M*_*nm*1_ and *M*_*nm*2_ can describe the time course of the BOLD response in the human visual cortex and correctly predict the response to several variations of the original stimulus not present in the estimation data. In contrast, the individual hypotheses *M*_*m*_ and *M*_*n*_ cannot alone describe the BOLD response in a realistic manner.

Systems biology opens the door to a new type of fMRI analysis, which is firmly based in the physiology of the neurovascular coupling behind the measured signal. Systems biology also gives us the opportunity to obtain information about neural activation beyond what we can measure and may thereby help deepen our understanding of the complex system that is the brain.

## Supporting Information

S1 Appendix The S1 Appendix contains interaction graphs, equations and parameter values for all models presented in this article. It also contains graphs showing the fit of the models *M*_*m*1_ and *M*_*n*2_ as well as the simulated glucose behaviour in the model *M*_*nm*1_.
(PDF)

S2 Appendix The S2 Appendix contains the BOLD response time series (group mean and SE) from all experiments used in this manuscript. (TXT)
