# Supplementary material for: Mechanistic Mathematical Modeling Tests Hypotheses of the Neurovascular Coupling in fMRI
Source: PLoS Comput Biol. 2016 Jun 16;12(6):e1004971. doi: 10.1371/journal.pcbi.1004971 (PMC4911100; doi:10.1371/journal.pcbi.1004971)
Supplement: S1 Appendix — It also contains graphs showing the fit of the models Mm1 and Mn2 as well as the simulated glucose behaviour in the model Mnm1. (PDF) [file pcbi.1004971.s001.pdf]

Appendix / Supplementary material

When using the material in this appendix, please cite Lundengård et al. "Mechanistic mathematical modeling tests hypotheses of the neurovascular coupling in fMRI", PLOS Computational, 2016.
DOI: 10.1371/journal.pcbi.1004971.

All models have been uploaded to the model archive biomodels.net, where the model and simulation files can be downloaded.

All models and parameter sets have the same name in the appendix as they have in the article. All steady state values were calculated by simulating the model until steady state has been reached (1000s).

1 The metabolic model M_m

1.1 M_{m1}

The metabolic model M_{m1} assumes that the only mechanism that controls the shape of the BOLD response is that the blood vessels increase the blood flow in response to a lack of oxygen during the stimulus.

1.1.1 States and reactions

State	Interpretation
$\frac{d(stimulus)}{dt} = 0$	Stimulus input signal
$\frac{d(oHb)}{dt} = v1_b - v1_f + v_{inoHb} - v_{outoHb}$	Change in oxyhemoglobin level
$\frac{d(dHb)}{dt} = v1_f - v1_b + v_{indHb} - v_{outdHb}$	Change in deoxyhemoglobin level
$\frac{d(O_2)}{dt} = v1_f - v1_b - v_{basal} \times proportion_1 - v_{stim} \times proportion_2 + v_{inO_2} - v_{outO_2}$	Change in oxygen level
$\frac{d(glucose)}{dt} = v_{inG} - v_{outG} - v_{basal} - v_{stim}$	Change in glucose level
$\frac{d(inputDelay)}{dt} = input_1 - v_{ID}$	Delay state
$\frac{d(oxygenFbDelay)}{dt} = v_{toOFBD} - v_{OFBD}$	Delay state
$\frac{d(oxygenFbDelay_2)}{dt} = v_{OFBD} - v_{OFBD2}$	Delay state
$\frac{d(oxygenFbDelay_3)}{dt} = v_{toOFBD2} - v_{OFBD3}$	Delay state
$\frac{d(oxygenfeedback)}{dt} = v_{OFBD3} - v_{vOFB}$	Oxygen feedback to the blood flow

Reaction	Interpretation
$v1_f = k1_f \times oHb$<br><br>$v1_b = k1_b \times dHb \times O_2$	Rate of releasing oxyhemoglobin into oxygen and deoxyhemoglobin<br><br>Rate of binding oxygen and deoxyhemoglobin into oxyhemoglobin
$v_{inoHb} = oHb_{body} \times k_{flow}$<br>$v_{outoHb} = oHb \times k_{flow}$<br><br>$v_{indHb} = dHb_{body} \times k_{flow}$<br>$v_{outdHb} = dHb \times k_{flow}$<br><br>$v_{inG} = G_{body} \times k_{flow}$<br>$v_{outG} = glucose \times k_{flow}$<br><br>$v_{inO_2} = O_{2body} \times k_{flow}$<br>$v_{outO_2} = O_2 \times k_{flow}$	Oxyhemoglobin influx<br>Oxyhemoglobin outflux<br><br>Deoxyhemoglobin influx<br>Deoxyhemoglobin outflux<br>Glucose influx<br>Glucose outflux<br><br>Oxygen influx<br>Oxygen outflux
$v_{basal} = k_{basal} \times O_2^{proportion_1} \times glucose$<br>$v_{stim} = inputDelay_5 \times O_2^{proportion_2} \times glucose$	Basal metabolism<br>Metabolism during stimulation
$input_1 = k_{metabolic} \times stimulus$<br>$v_{ID} = k_{ID} \times input_{Delay}$	Delay state reactions
$v_{toOFBD} = O_{2Dbody} \times glucose$<br>$v_{OFBD} = oxygen_{FBdelay} \times k_{OFBD}$<br>$v_{OFBD2} = oxygen_{FBdelay2} \times k_{OFBD2}$<br>$v_{OFBD3} = oxygen_{FBdelay3} \times k_{OFBD3}$<br>$v_{OFB} = oxygenfeedback \times k_{OFB}$	Delay state reactions

1.1.2 Variables

Variable name	Variable unit	Variable value	Interpretation
k_{flow}	1/s	$\frac{kflow_{O_2}}{km + oxygenfeedback}$	Blood flow
G_{body}	amount	100	Glucose in arterial blood
O_{2body}	amount	100	Oxygen in arterial blood
oHb_{body}	amount	100	Oxygenated hemoglobin in arterial blood
dHb_{body}	amount	100	Deoxygenated hemoglobin in arterial blood
ŷ	unitless	$\frac{k_y \times oHb}{dHb}$	Output signal

1.1.3 Parameters and parameter values

Parameters and parameter values for the metabolic feedback model M_{m1} used in Fig. A.

**Parameter name**	**Parameter unit**	**Parameter value**
$k1_f$	1/s	14049.3847
$k1_b$	1/(amount×s)	18645.2545
k_{basal}	1/(amount×s)	0.5064
$kflow_{O2}$	amount/s	3.0207
k_y	unitless	12514.7912
$k_{metabolic}$	1/s	1907.5170
k_m	amount	604.8624
proportion1	oxygen/glucose metabolised	0.9222
proportion2	oxygen/glucose metabolised	0.1632
k_{ID}	1/s	6250.7308
O_{2Dbody}	1/s	29.8642
k_{OFBD}	1/s	5.5231
k_{OFBD2}	1/s	16208.1574
k_{OFBD3}	1/s	16.3427
k_{OFB}	1/s	3189.4025

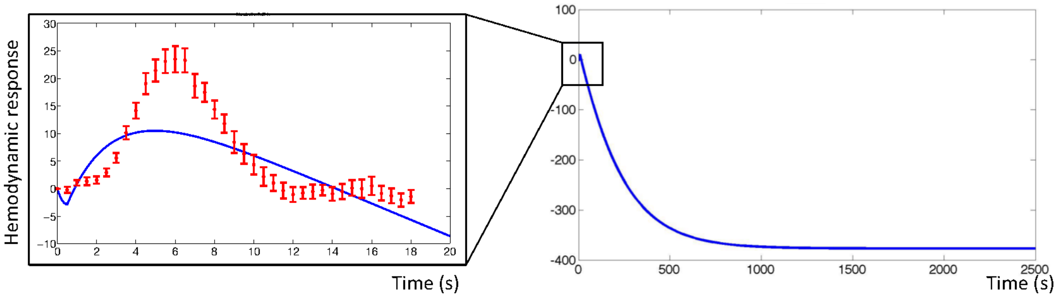

Fig A: Oxygen controls the feedback in the metabolic model M_{m1}. Red dots = data mean and SE, blue line = model simulation.

1.2 M_{m2}

The metabolic model M_{m2} assumes that the only mechanism that controls the shape of the BOLD response is that the blood vessels increase the blood flow in response to a lack of glucose during the stimulus.

1.2.1 States and reactions

State	Interpretation	Steady State value p_1	Steady State value p_2	Steady State value p_3
$\frac{d(stimulus)}{dt} = 0$	Stimulus input signal	0	0	0
$\frac{d(oHb)}{dt} = v1_b - v1_f + v_{inoHb} - v_{outoHb}$	Change in oxyhemoglobin level	107.10	107.10	107.1
$\frac{d(dHb)}{dt} = v1_f - v1_b + v_{indHb} - v_{outdHb}$	Change in deoxyhemoglobin level	92.90	92.90	92.9
$\frac{d(O_2)}{dt} = v1_f - v1_b - v_{basal} \times proportion_1 - v_{stim} \times proportion_2 + v_{inO_2} - v_{outO_2}$	Change in oxygen level	0.52	0.52	0.52
$\frac{d(glucose)}{dt} = v_{inG} - v_{outG} - v_{basal} - v_{stim}$	Change in glucose level	29.80	29.80	29.8
$\frac{d(inputDelay)}{dt} = input_1 - v_{ID}$	Delay state	4.65×10^{-20}	-1.52×10^{-17}	-2.08×10^{-18}
$\frac{d(inputDelay_2)}{dt} = v_{ID} - v_{ID2}$	Delay state	4.63×10^{-20}	-1.51×10^{-17}	-2.07×10^{-18}
$\frac{d(inputDelay_3)}{dt} = v_{ID2} - v_{ID3}$	Delay state	4.70×10^{-20}	-1.54×10^{-17}	-2.1×10^{-18}
$\frac{d(inputDelay_4)}{dt} = v_{ID3} - v_{ID4}$	Delay state	4.59×10^{-20}	-1.50×10^{-17}	-2.05×10^{-18}
$\frac{d(inputDelay_5)}{dt} = v_{ID4} - v_{ID5}$	Delay state	1.66×10^{-23}	-5.41×10^{-21}	-7.4×10^{-22}
$\frac{d(glucoseFbDelay)}{dt} = v_{toGFBD} - v_{GFBD}$	Delay state	0.16	0.16	0.16
$\frac{d(glucosefeedback)}{dt} = v_{GFBD} - v_{GFB}$	Glucose feedback to the blood flow	0.25	0.25	0.25

Reaction	Interpretation
$v_{inoHb} = oHb_{body} \times k_{flow}$<br>$v_{outoHb} = oHb \times k_{flow}$<br><br>$v_{indHb} = dHb_{body} \times k_{flow}$<br>$v_{outdHb} = dHb \times k_{flow}$<br><br>$v_{inG} = G_{body} \times k_{flow}$<br>$v_{outG} = glucose \times k_{flow}$<br><br>$v_{inO_2} = O_{2body} \times k_{flow}$<br>$v_{outO_2} = O_2 \times k_{flow}$	Oxyhemoglobin influx<br>Oxyhemoglobin outflux<br><br>Deoxyhemoglobin influx<br>Deoxyhemoglobin outflux<br>Glucose influx<br>Glucose outflux<br><br>Oxygen influx<br>Oxygen outflux
$v_{basal} = k_{basal} \times O_2^{proportion_1} \times glucose$<br>$v_{stim} = inputDelay_5 \times O_2^{proportion_2} \times glucose \times k_{i2}$	Basal metabolism<br>Metabolism during stimulation
$input_1 = k_{metabolic} \times stimulus$<br>$v_{ID} = k_{ID} \times input_{Delay}$	Delay state reactions
$v_{toGFBD} = GD_{body} \times glucose$<br>$v_{GFBD} = glucose_{FBdelay} \times k_{GFBD}$<br>$v_{GFB} = glucosefeedback \times k_{GFB}$	Delay state reactions
$v_{ID2} = input_{Delay2} \times k_{ID2}$<br>$v_{ID3} = input_{Delay3} \times k_{ID3}$<br>$v_{ID4} = input_{Delay4} \times k_{ID4}$<br>$v_{ID5} = input_{Delay5} \times k_{ID5}$	Delay state reactions

1.2.2 Variables

Variable name	Variable unit	Variable value	Interpretation
k_{flow}	1/s	$\frac{kflow_{glucose}}{km + glucosefeedback}$	Blood flow
G_{body}	amount	100	Glucose in arterial blood
GD_{body}	amount	100	Glucose feedback delay
O_{2body}	amount	100	Oxygen in arterial blood
oHb_{body}	amount	100	Oxygenated hemoglobin in arterial blood
dHb_{body}	amount	100	Deoxygenated hemoglobin in arterial blood
ŷ	unitless	$\frac{k_y \times oHb}{dHb}$	Output signal

1.2.3 Parameters and parameter values

Parameter sets for model M_{m2} used in Fig. 5 B-D. Proportion1 and proportion2 (marked in bold) control the proportion of aerobic and anaerobic metabolism during basal conditions vs. during stimulation.

**Parameter names**	**Parameter unit**	**p_1 $(CMR_{O2}/CMR_{glu})_{basal} = (CMR_{O2}/CMR_{glu})_{stimuli}$**	**p_2 $(CMR_{O2})_{stimuli} = 0$**	**p_3 $(CMR_{O2}/CMR_{glu})_{basal} > (CMR_{O2}/CMR_{glu})_{stimuli}$**
$k1_f$	1/s	627.1792	627.1792	627.1792
$k1_b$	1/(amount×s)	1381.9932	1381.9932	1381.9932
k_{basal}	1/(amount×s)	5.0587	5.0587	5.0587
$kflow_{glucose}$	amount/s	102.6292	102.6292	102.6292
k_y	unitless	2905.5532	2905.5532	2905.5532
$k_{metabolic}$	1/s	189.3795	189.3795	189.3795
k_m	amount	111.8459	111.8459	111.8459
k_{i2}	$1/(amount^2 \times s)$	11.67404	11.67404	11.67404
proportion1	oxygen/glucose metabolised	**1.3159**	**1.3159**	**1.3159**
proportion2	oxygen/glucose metabolised	**1.3159**	**0**	**0.6304**
k_{ID}	1/s	0.9575	0.9575	0.9575
k_{GFBD}	1/s	18641.1377	18641.137 7	18641.1377
k_{GFB}	1/s	11921.2175	11921.217 5	11921.2175
k_{ID2}	1/s	0.9613	0.9613	0.9613
k_{ID3}	1/s	0.9484	0.9484	0.9484
k_{ID4}	1/s	0.9703	0.9703	0.9703
k_{ID5}	1/s	2687.7052	2687.7052	2687.7052

1.3 M_{m3}

The metabolic model M_{m3} assumes that the only mechanism that controls the shape of the BOLD response is that the blood vessels increase the blood flow in response to a lack of glucose during the stimulus. The delay states are placed in the glucose feedback, not between stimulus and metabolism.

1.3.1 Interaction graph

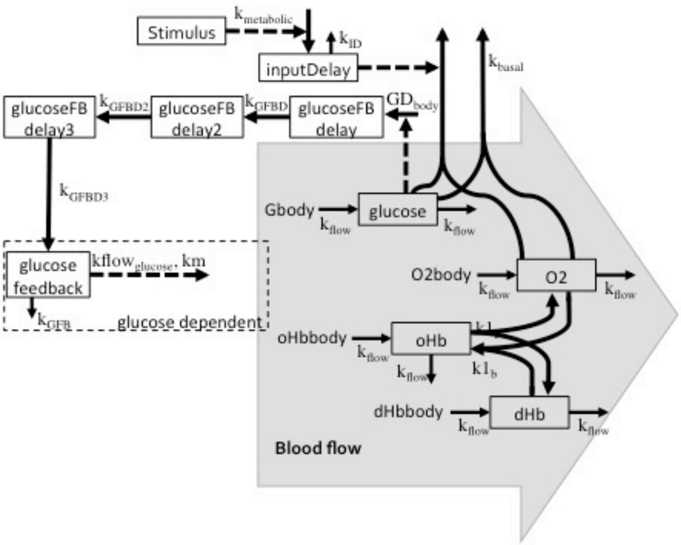

Fig. B: Interaction graph of the metabolic feedback model M_{m3}.
Interaction graphs of the metabolic feedback model and the neurotransmitter feed-forward model.

Whole squares = states, dashed squares = variables (summed from states), whole arrows = transformations, dashed arrows = interactions, green area = astrocyte, blue area = neuron, grey area = blood. All states starting with S and a number are delay states. Stimulus is the input to the model. Gbody, O2body, oHbbody, dHbbody, and PL are variables. Stimulus = input signal. oHb and dHb are oxyhemoglobin and deoxyhemoglobin, respectively.

1.3.2 States and reactions

State	Interpretation	Steady State value p4	Steady State value p5
$\frac{d(stimulus)}{dt} = 0$	Stimulus input signal	0	0
$\frac{d(oHb)}{dt} = v1_b - v1_f + v_{inoHb} - v_{outoHb}$	Change in oxyhemoglobin level	1.84	16.15
$\frac{d(dHb)}{dt} = v1_f - v1_b + v_{indHb} - v_{outdHb}$	Change in deoxyhemoglo bin level	18.16	3.85
$\frac{d(O_2)}{dt} = v1_f - v1_b - v_{basal} \times proportion_1 - v_{stim} \times proportion_2 + v_{inO_2} - v_{outO_2}$	Change in oxygen level	0.12	0.0018
$\frac{d(glucose)}{dt} = v_{inG} - v_{outG} - v_{basal} - v_{stim}$	Change in glucose level	1.41	5.60
$\frac{d(inputDelay)}{dt} = input_1 - v_{ID}$	Delay state	$-2.11*10^{-14}$	$-8.12*10^{-15}$
$\frac{d(glucoseFbDelay)}{dt} = v_{toGFBD} - v_{GFBD}$ $\frac{d(glucoseFbDelay_2)}{dt} = v_{GFBD} - v_{GFBD2}$ $\frac{d(glucoseFbDelay_3)}{dt} = v_{GFBD2} - v_{GFBD3}$ $\frac{d(glucosefeedback)}{dt} = v_{GFBD3} - v_{GFB}$	Delay states	0.18	3897.12

Reaction	Interpretation
$v1_f = k1_f \times oHb$<br><br>$v1_b = k1_b \times dHb \times O_2$	Rate of releasing oxyhemoglobin into oxygen and deoxyhemoglobin<br><br>Rate of binding oxygen and deoxyhemoglobin into oxyhemoglobin
$v_{inoHb} = oHb_{body} \times k_{flow}$<br>$v_{outoHb} = oHb \times k_{flow}$<br><br>$v_{indHb} = dHb_{body} \times k_{flow}$<br>$v_{outdHb} = dHb \times k_{flow}$<br><br>$v_{inG} = G_{body} \times k_{flow}$<br>$v_{outG} = glucose \times k_{flow}$<br><br>$v_{inO_2} = O_{2body} \times k_{flow}$<br>$v_{outO_2} = O_2 \times k_{flow}$	Oxyhemoglobin influx<br>Oxyhemoglobin outflux<br><br>Deoxyhemoglobin influx<br>Deoxyhemoglobin outflux<br><br>Glucose influx<br>Glucose outflux<br><br>Oxygen influx<br>Oxygen outflux
$v_{basal} = k_{basal} \times O_2^{proportion_1} \times glucose$<br>$v_{stim} = inputDelay \times O_2^{proportion_2} \times glucose$	Basal metabolism<br><br>Metabolism during stimulation
$input_1 = k_{metabolic} \times stimulus$<br>$v_{ID} = k_{ID} \times input_{Delay}$	Delay state reactions
$v_{toGFBD} = GD_{body} \times glucose$<br>$v_{GFBD} = glucose_{FBdelay} \times k_{GFBD}$<br>$v_{GFBD2} = glucose_{FBdelay2} \times k_{GFBD2}$<br>$v_{GFBD3} = glucose_{FBdelay3} \times k_{GFBD3}$<br>$v_{GFB} = glucosefeedback \times k_{GFB}$	Delay state reactions

1.3.3 Variables

Variable name	Variable unit	Variable value	Interpretation
k_{flow}	1/s	$\frac{kflow_{glucose}}{km + glucosefeedback}$	Blood flow
G_{body}	amount	100	Glucose in arterial blood
O_{2body}	amount	100	Oxygen in arterial blood
oHb_{body}	amount	100	Oxygenated hemoglobin in arterial blood
dHb_{body}	amount	100	Deoxygenated hemoglobin in arterial blood
ŷ	unitless	$\frac{k_y \times oHb}{dHb}$	Output signal

1.3.4 Parameters and parameter values

Parameter sets for model M_{m3} used in Fig. 5 G-J.

**Parameter names**	**Parameter unit**	**p_4 Only undershoot**	**p_5 Only initial dip**
$k1_f$	1/s	1.3952	8.8366
$k1_b$	1/(amount×s)	0.0011	19957.2824
k_{basal}	1/(amount×s)	174.5880	41.8601
$kflow_{glucose}$	amount/s	2438.7260	818.7605
k_y	unitless	297.9752	2.7266
$k_{metabolic}$	1/s	19943.3114	19852.9448
km	amount	3.3201	0.1918
proportion1	oxygen/glucose metabolised	2.1006	0.8738
proportion2	oxygen/glucose metabolised	0.0011	0.1102
k_{ID}	1/s	7.0830	1.4594
GD_{body}	1/s	2603.4638	545.2871
k_{GFBD}	1/s	19932.2693	0.7838
k_{GFBD2}	1/s	0.6959	0.8452
k_{GFBD3}	1/s	0.6676	4916.7131
k_{GFB}	1/s	0.4752	0.8249

Cost: p_4 = 18.02, p_5 = 60.84.

2 The Neurotransmitter model M_n

2.1 M_{n1}

The neurotransmitter model M_{n1} assumes that the mechanism that controls the shape of the BOLD response is the vessel response to signaling substances released by neurons and astrocytes in response to a stimulus.

2.1.1 Interaction graph

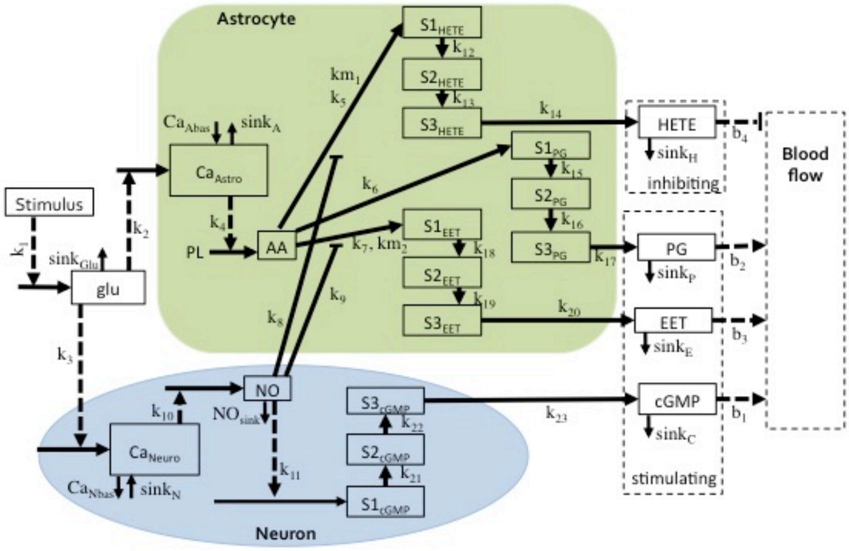

Fig. C: Interaction graph of the neurotransmitter feed-forward model. The neurotransmitter feed-forward hypothesis is described in more detail in Attwell 2010. Whole squares = states, dashed squares = variables (summed from states), whole arrows = transformations, dashed arrows = interactions, green area = astrocyte, blue area = neuron. All states starting with S and a number are delay states. Stimulus is the input to the model. Calcium neuron and calcium Astrocyte = calcium ion (Ca^{2+}) level in the cell, NO = nitric oxide, cGMP = cyclic guanosine monophosphate, AA = arachidonic acid, EET = epoxyeicosatrienoic acids, PG = prostaglandins and HETE = hydroxyeicosatetraeonic acid (20-HETE).

2.1.2 States and reactions

State	Interpretation	Steady State value p_6
$\frac{d(Stimulus)}{dt} = 0$	Stimulus input signal	0
$\frac{d(glu)}{dt} = in - glu_{sink}$	Glutamate release in the synaptic cleft	0
$\frac{d(Ca_{Astro})}{dt} = Glutamate_A - calcium_{Astro1} + Ca_{Abas}$	Calcium influx in the astrocyte	1.45
$\frac{d(AA)}{dt} = calcium_{Astro2} - (AA_{HETE} + AA_{PG} + AA_{EET})$	Change in AA level	768.92
$\frac{d(Ca_{Neuro})}{dt} = Glutamate_N - calcium_{Neuro1} + Ca_{Nbas}$	Calcium influx in the neuron	0.78
$\frac{d(NO)}{dt} = calcium_{Neuro2} - sink_{NO}$	Change in NO level	0.72
$\frac{d(HETE)}{dt} = v3_{HETE} - HETE_{sink}$<br>$\frac{d(S1_{HETE})}{dt} = AA_{HETE} - v1_{HETE}$<br>$\frac{d(S2_{HETE})}{dt} = v1_{HETE} - v2_{HETE}$<br>$\frac{d(S3_{HETE})}{dt} = v2_{HETE} - v3_{HETE}$	HETE effecting the blood vessels<br><br>Delay states	736.81<br>237.40<br>593.44<br>293.89
$\frac{d(PG)}{dt} = v3_{PG} - PG_{sink}$<br>$\frac{d(S1_{PG})}{dt} = AA_{PG} - v1_{PG}$<br>$\frac{d(S2_{PG})}{dt} = v1_{PG} - v2_{PG}$<br>$\frac{d(S3_{PG})}{dt} = v2_{PG} - v3_{PG}$	PG effecting the blood vessels<br><br>Delay states	740.05<br>708.47<br>729.05<br>723.29
$\frac{d(EET)}{dt} = v3_{EET} - EET_{sink}$<br>$\frac{d(S1_{EET})}{dt} = AA_{EET} - v1_{EET}$<br>$\frac{d(S2_{EET})}{dt} = v1_{EET} - v2_{EET}$<br>$\frac{d(S3_{EET})}{dt} = v2_{EET} - v3_{EET}$	EET effecting the blood vessels<br><br>Delay states	722.52<br>851.13<br>664.34<br>707.88
$\frac{d(cGMP)}{dt} = v3_{cGMP} - cGMP_{sink}$<br>$\frac{d(S1_{cGMP})}{dt} = AA_{cGMP} - v1_{cGMP}$<br>$\frac{d(S2_{cGMP})}{dt} = v1_{cGMP} - v2_{cGMP}$<br>$\frac{d(S3_{cGMP})}{dt} = v2_{cGMP} - v3_{cGMP}$	cGMP effecting the blood vessels<br><br>Delay states	0.78<br>0.71<br>0.81<br>0.67

Reactions	Interpretation
$in = k_1 \times Stimulus$<br>$glu_{Sink} = glu \times sink_{Glu}$	Stimulus input<br>Glucose breakdown and reuptake
$Glutamate_A = k_2 \times glu$<br>$calcium_{Astro1} = Ca_{Astro} \times sink_A$<br>$calcium_{Astro2} = PL \times Ca_{Astro} \times k_4$	Calcium influx in the astrocyte<br>Calcium outflux in the astrocyte<br>Calcium induced AA
$AA_{HETE} = k_5 \times \frac{AA}{(km_1 + k_8 \times NO)}$<br>$AA_{PG} = k_6 \times AA$<br>$AA_{EET} = k_7 \times \frac{AA}{(km_2 + k_9 \times NO)}$	AA turning into HETE<br>AA turning into PG<br>AA turning into EET
$Glutamate_N = k_3 \times glu$<br>$calcium_{Neuro1} = Ca_{Neuro} \times sink_N$<br>$calcium_{Neuro2} = k_{10} \times Ca_{Neuro}$	Calcium influx in the neuron<br>Calcium outflux in the neuron<br>Calcium induced NO
$NO_{cGMP} = k_{11} \times NO$<br>$sink_{NO} = NO_{sink} \times NO$	NO induced cGMP<br>NO breakdown
$v1_{HETE} = k_{12} \times S1_{HETE}$<br>$v2_{HETE} = k_{13} \times S2_{HETE}$<br>$v3_{HETE} = k_{14} \times S3_{HETE}$<br>$HETE_{sink} = HETE \times sink_H$	Delay state reactions<br>HETE breakdown
$v1_{PG} = k_{15} \times S1_{PG}$<br>$v2_{PG} = k_{16} \times S2_{PG}$<br>$v3_{PG} = k_{17} \times S3_{PG}$<br>$PG_{sink} = PG \times sink_P$	Delay state reactions<br>PG breakdown
$v1_{EET} = k_{18} \times S1_{EET}$<br>$v2_{EET} = k_{19} \times S2_{EET}$<br>$v3_{EET} = k_{20} \times S3_{EET}$<br>$EET_{sink} = EET \times sink_E$	Delay state reactions<br>EET breakdown
$v1_{cGMP} = k_{21} \times S1_{cGMP}$<br>$v2_{cGMP} = k_{22} \times S2_{cGMP}$<br>$v3_{cGMP} = k_{23} \times S3_{cGMP}$<br>$cGMP_{sink} = cGMP \times sink_c$	Delay state reactions<br>cGMP breakdown

2.1.3 Variables

Variable name	Variable unit	Variable value
Stimulating	unitless	$b_1 \times cGMP + b_2 \times PG + b_3 \times EET$
Inhibiting	unitless	$b_4 \times HETE$
ŷ	unitless	$b_1 \times cGMP + b_2 \times PG + b_3 \times EET - b_4 \times HETE$

2.1.4 Parameters and parameter values

Parameter sets for model M_{n1} used in Fig. 6A.

Parameter name	Parameter unit	Parameter Value p_6
k_1	1/s	0.5589
k_2	1/s	0.053036
k_3	1/s	3.3634
k_4	1/(amount×s)	1.6775
k_5	amount/s	0.43467
k_6	1/s	5.892×10^7
k_7	amount/s	1.7891
k_8	unitless	1.0276
k_9	unitless	2.8976
k_{10}	1/s	1.0993
k_{11}	1/s	4.8221×10^5
k_{12}	1/s	6.3353×10^8
k_{13}	1/s	0.87781
k_{14}	1/s	1.0343
k_{15}	1/s	2.1986
k_{16}	1/s	0.8836
k_{17}	1/s	1.2209
k_{18}	1/s	0.6518
k_{19}	1/s	0.9190
k_{20}	1/s	7.5887×10^7
k_{21}	1/s	2.5067
k_{22}	1/s	1.7921
k_{23}	1/s	1.5562×10^5
km_1	amount	5.5066×10^6
km_2	amount	0.0433
b_1	1/amount	2.7762
b_2	1/amount	1.299
b_3	1/amount	0.8634
b_4	1/amount	1.0201×10^7
Ca_{Abas}	amount/s	3.3823×10^7
Ca_{Nbas}	amount/s	1.4943
$sink_{Glu}$	1/s	0.5318
$sink_A$	1/s	1.3426
$sink_N$	1/s	0.9493
$sink_H$	1/s	5.7961×10^7
$sink_P$	1/s	0.9134

$sink_E$	1/s	0.7554
$sink_c$	1/s	6.7692×10^5
NO_{sink}	1/s	1.0735
PL	amount/s	945.52

Cost: 5.76.

2.2 M_{n2}

Minimized version of the neurotransmitter model M_{n1}. The main mechanism is the balance between the vasoconstricting and the vasodilating arm of the model structure.

The states, variables and parameters of this model do not have a biological interpretation.

2.2.1 Interaction graph

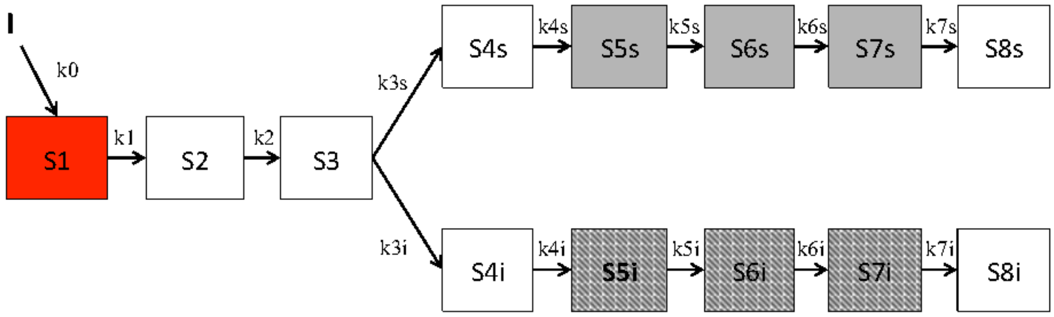

Fig. D. Interaction graph of the minimized neurotransmitter feed-forward model. Filled grey squares = vasodilation states, checkered grey squares = vasoconstriction states.

2.2.2 Fit to data

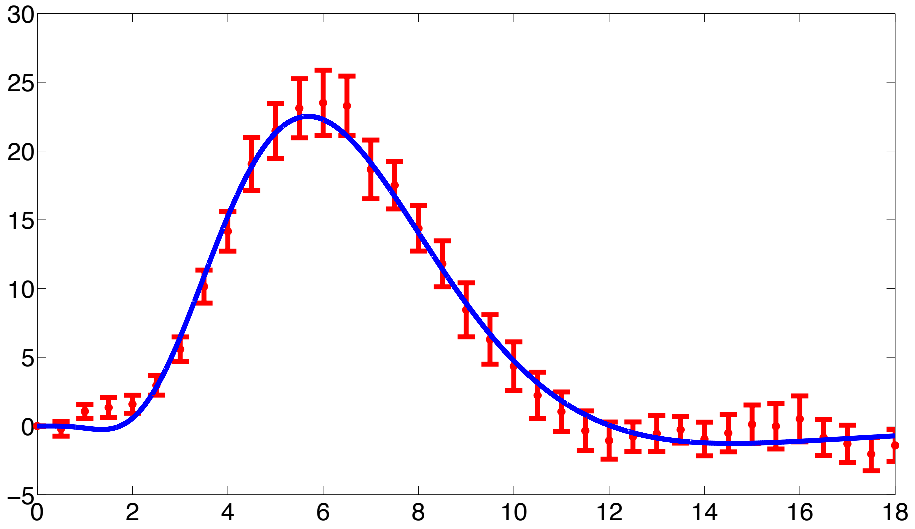

Fig E: Fit of the minimal model M_{n2} to data. Red dots = data mean and SE, blue line = model simulation.

2.2.3 States and reactions

State equation	**Steady State values best fit intensity experiment**
$\frac{d(I)}{dt} = 0$	3.86×10^{-21}
$\frac{d(S1)}{dt} = I\times k_0 - S1\times k_1$	-1.88×10^{-14}
$\frac{d(S2)}{dt} = S1\times k_1 - S2\times k_2$	-3.71×10^{-14}
$\frac{d(S3)}{dt} = S2\times k_2 - S3\times(k_{3s} + k_{3i})$	-2.30×10^{-14}
$\frac{d(S4_s)}{dt} = S3\times k_{3s} - S4\times k_{4s}$	-2.54×10^{-13}
$\frac{d(S5_s)}{dt} = S4\times k_{4s} - S5\times k_{5s}$	-3.75×10^{-13}
$\frac{d(S6_s)}{dt} = S5\times k_{5s} - S6\times k_{6s}$	-2.10×10^{-16}
$\frac{d(S7_s)}{dt} = S6\times k_{6s} - S7\times k_{7s}$	-1.42×10^{-12}
$\frac{d(S8_s)}{dt} = S7_s\times k_{7s}$	0.57
$\frac{d(S4_i)}{dt} = S3\times k_{3i} - S4\times k_{4i}$	-4.93×10^{-14}
$\frac{d(S5_i)}{dt} = S4\times k_{4i} - S5\times k_{5i}$	2.46×10^{-12}
$\frac{d(S6_i)}{dt} = S5\times k_{5i} - S6\times k_{6i}$	5.29×10^{-13}
$\frac{d(S7_i)}{dt} = S6\times k_{6i} - S7\times k_{7i}$	6.25×10^{-18}
$\frac{d(S8_i)}{dt} = S7_i\times k_{7i}$	0.43

2.2.4 Variables

**Variable name**	**Variable unit**	**Variable value**
Stimulating	unitless	$S5_s + S6_s + S7_s$
Inhibitory	unitless	$S5_i+S6_i+S7_i$
ŷ	unitless	$k_y\times (S5_s-S5_i+S6_s-S6_i+S7_s-S7_i)$

2.2.5 Parameters and parameter values

Parameter sets for model M_{n2} used in Fig. D.

**Parameter names**	**Parameter unit**	**Parameter values best fit intensity experiment**
k_y	unitless	13467
k_0	1/s	0.1034
k_1	1/s	0.6245
k_2	1/s	0.9430
k_{3s}	1/s	1.2261
k_{4s}	1/s	0.7059
k_{5s}	1/s	1.0579
k_{6s}	1/s	1889.7
k_{7s}	1/s	0.7730
k_{3i}	1/s	0.9163
k_{4i}	1/s	1.0627
k_{5i}	1/s	0.4454
k_{6i}	1/s	2.5427
k_{7i}	1/s	0.000021531

Cost: 29.2.

3 The feed-forward with metabolism model M_{nm}

3.1 M_{nm1} Model structure

The combined model M_{nm1} is a merge between the metabolic model M_{m3} and the neurotansmitter model M_{n1}. It assumes that the vessel response to signaling substances released by neurons and astrocytes in response to a stimulus controls the blood flow. The metabolism controls the balance of dHb and oHb. Therefore, both the metabolism and the intracellular signaling controls the shape of the BOLD response. The metabolism of O_2 is the main mechanism behind the intial dip while the blood flow controls the peak and the post-peak undershoot.

3.1.1 Interactiongraph

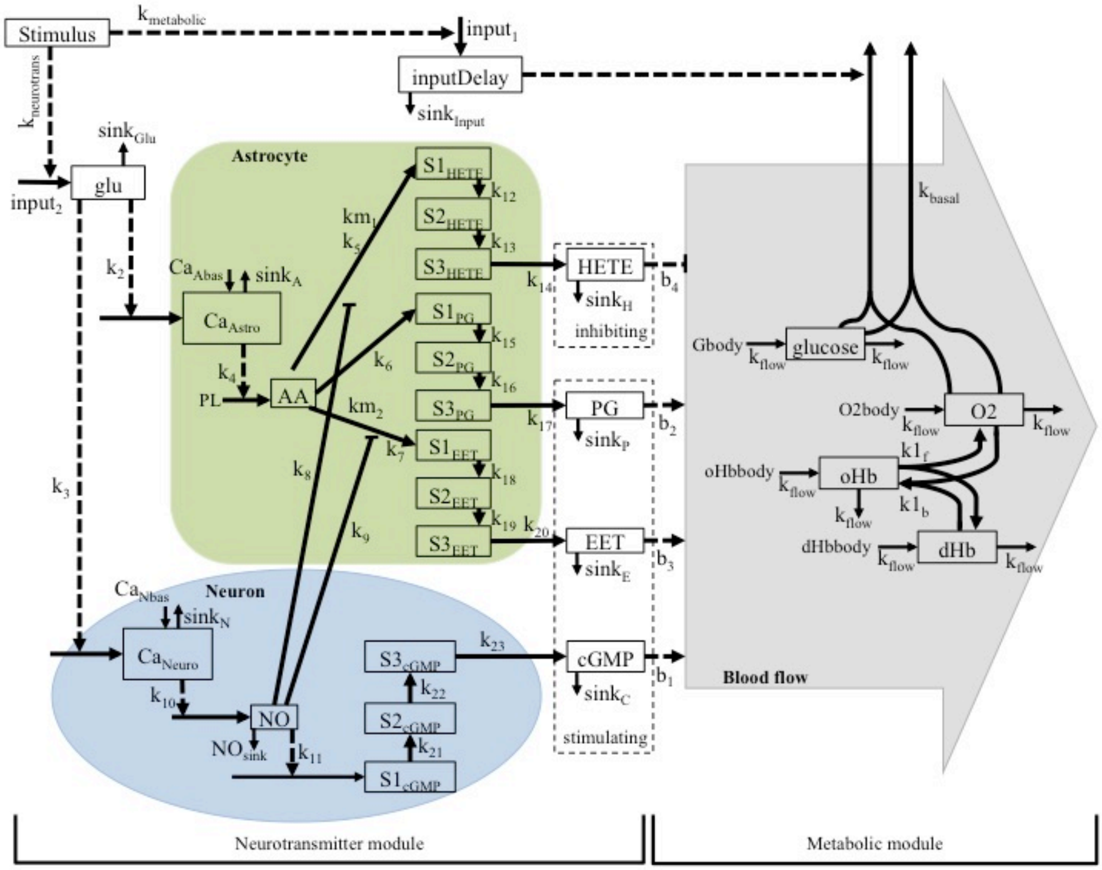

Fig. F: Interaction graph of the combined model M_{mn1}. All abbreviations and names as declared above.

3.1.2 States and reactions

State	Interpretation	Steady State value p7	Steady State value p9
$\frac{d(stimulus)}{dt} = 0$	Stimulus input signal	0	0
$\frac{d(oHb)}{dt} = v1_b - v1_f + v_{inoHb} - v_{outoHb}$	Change in oxyhemoglobin level	5.54	7.21
$\frac{d(dHb)}{dt} = v1_f - v1_b + v_{indHb} - v_{outdHb}$	Change in deoxyhemoglobin level	14.45	12.79
$\frac{d(O_2)}{dt} = v1_f - v1_b - v_{basal} \times proportion_1 - v_{stim} \times proportion_2 + v_{inO_2} - v_{outO_2}$	Change in oxygen level	0.8	1.26
$\frac{d(glucose)}{dt} = v_{inG} - v_{outG} - v_{basal} - v_{stim}$	Change in glucose level	6.85	8.66
$\frac{d(inputDelay)}{dt} = input_1 - v_{ID}$	Delay state	5.54×10^{-17}	7.85×10^{-18}
$\frac{d(glu)}{dt} = input_2 - glu_{sink}$	Glutamate release in the synaptic cleft	1.54×10^{-17}	6.08×10^{-19}
$\frac{d(Ca_{Astro})}{dt} = Glutamate_A - calcium_{Astro1} + Ca_{Abas}$	Calcium influx in the astrocyte	8.67	2.15
$\frac{d(AA)}{dt} = calcium_{Astro2} - (AA_{HETE} + AA_{PG} + AA_{EET})$	Change in AA level	1433.15	36.56
$\frac{d(Ca_{Neuro})}{dt} = Glutamate_N - calcium_{Neuro1} + Ca_{Nbas}$	Calcium influx in the neuron	2885.39	2.52
$\frac{d(NO)}{dt} = calcium_{Neuro2} - sink_{NO}$	Change in NO level	127.69	0.09
$\frac{d(HETE)}{dt} = v3_{HETE} - HETE_{sink}$<br>$\frac{d(S1_{HETE})}{dt} = AA_{HETE} - v1_{HETE}$<br>$\frac{d(S2_{HETE})}{dt} = v1_{HETE} - v2_{HETE}$<br>$\frac{d(S3_{HETE})}{dt} = v2_{HETE} - v3_{HETE}$	HETE effecting the blood vessels<br><br>Delay states	81.99<br>1.084<br>95.37<br>58.49	38.65<br>25.23<br>41.52<br>32.79
$\frac{d(PG)}{dt} = v3_{PG} - PG_{sink}$<br>$\frac{d(S1_{PG})}{dt} = AA_{PG} - v1_{PG}$<br>$\frac{d(S2_{PG})}{dt} = v1_{PG} - v2_{PG}$<br>$\frac{d(S3_{PG})}{dt} = v2_{PG} - v3_{PG}$	PG effecting the blood vessels<br><br>Delay states	0.06<br>2.62<br>0.37<br>0.084	0.33<br>1.77<br>0.39<br>1.49

$\frac{d(EET)}{dt} = v3_{EET} - EET_{sink}$<br>$\frac{d(S1_{EET})}{dt} = AA_{EET} - v1_{EET}$<br>$\frac{d(S2_{EET})}{dt} = v1_{EET} - v2_{EET}$<br>$\frac{d(S3_{EET})}{dt} = v2_{EET} - v3_{EET}$	EET effecting the blood vessels<br><br>Delay states	31.99<br>39.64<br>34.05<br>31.84	21.10<br>41.98<br>18.74<br>28.36
$\frac{d(cGMP)}{dt} = v3_{cGMP} - cGMP_{sink}$<br>$\frac{d(S1_{cGMP})}{dt} = AA_{cGMP} - v1_{cGMP}$<br>$\frac{d(S2_{cGMP})}{dt} = v1_{cGMP} - v2_{cGMP}$<br>$\frac{d(S3_{cGMP})}{dt} = v2_{cGMP} - v3_{cGMP}$	cGMP effecting the blood vessels<br><br>Delay states	15.28<br>2.14<br>0.47<br>1.39	0.001<br>0.01<br>0.07<br>0.02

**Reactions**	**Interpretation**
$v1_f = k1_f \times oHb$<br><br>$v1_b = k1_b \times dHb \times O_2$	Rate of releasing oxyhemoglobin into oxygen and deoxyhemoglobin<br><br>Rate of binding oxygen and deoxyhemoglobin into oxyhemoglobin
$v_{inoHb} = oHb_{body} \times k_{flow}$<br>$v_{outoHb} = oHb \times k_{flow}$<br><br>$v_{indHb} = dHb_{body} \times k_{flow}$<br>$v_{outdHb} = dHb \times k_{flow}$<br><br>$v_{inG} = G_{body} \times k_{flow}$<br>$v_{outG} = glucose \times k_{flow}$<br><br>$v_{inO_2} = O_{2body} \times k_{flow}$<br>$v_{outO_2} = O_2 \times k_{flow}$	Oxyhemoglobin influx<br>Oxyhemoglobin outflux<br><br>Deoxyhemoglobin influx<br>Deoxyhemoglobin outflux<br><br>Glucose influx<br>Glucose outflux<br><br>Oxygen influx<br>Oxygen outflux
$v_{basal} = k_{basal} \times O_2^{proportion_1} \times glucose$<br>$v_{stim} = inputDelay \times O_2^{proportion_2} \times glucose$	Basal metabolism<br>Metabolism during stimulation
$input_1 = k_{metabolic} \times stimulus$<br>$v_{ID} = sink_{Input} \times input_{Delay}$	Stimulus input to the metabolic module<br>Delay state reaction
$input_2 = k_{neurotrans} \times stimulus$<br><br>$glu_{Sink} = sink_{Glu} \times glu$	Stimulus input to the neurotransmitter module<br>Glucose breakdown and reuptake
$Glutamate_A = k_2 \times glu$<br>$calcium_{Astro1} = Ca_{Astro} \times sink_A$<br>$calcium_{Astro2} = PL \times Ca_{Astro} \times k_4$	Calcium influx in the astrocyte<br>Calcium outflux in the astrocyte<br>Calcium induced AA
$AA_{HETE} = k_5 \times \frac{AA}{(km_1 + k_8 \times NO)}$<br>$AA_{PG} = k_6 \times AA$<br>$AA_{EET} = k_7 \times \frac{AA}{(km_2 + k_9 \times NO)}$	AA turning into HETE<br><br>AA turning into PG<br><br>AA turning into EET
$Glutamate_N = k_3 \times glu$<br>$calcium_{Neuro1} = Ca_{Neuro} \times sink_N$<br>$calcium_{Neuro2} = k_{10} \times Ca_{Neuro}$	Calcium influx in the neuron<br>Calcium outflux in the neuron<br>Calcium induced NO
$NO_{cGMP} = k_{11} \times NO$<br>$sink_{NO} = NO_{sink} \times NO$	NO induced cGMP<br>NO breakdown
$v1_{HETE} = k_{12} \times S1_{HETE}$<br>$v2_{HETE} = k_{13} \times S2_{HETE}$<br>$v3_{HETE} = k_{14} \times S3_{HETE}$<br>$HETE_{sink} = HETE \times sink_H$	Delay state reactions<br><br>HETE breakdown
$v1_{PG} = k_{15} \times S1_{PG}$<br>$v2_{PG} = k_{16} \times S2_{PG}$<br>$v3_{PG} = k_{17} \times S3_{PG}$<br>$PG_{sink} = PG \times sink_P$	Delay state reactions<br><br>PG breakdown
$v1_{EET} = k_{18} \times S1_{EET}$<br>$v2_{EET} = k_{19} \times S2_{EET}$	Delay state reactions

$v3_{EET} = k_{20} \times S3_{EET}$<br>$EET_{sink} = EET \times sink_E$	EET breakdown
$v1_{cGMP} = k_{21} \times S1_{cGMP}$<br>$v2_{cGMP} = k_{22} \times S2_{cGMP}$<br>$v3_{cGMP} = k_{23} \times S3_{cGMP}$<br>$cGMP_{sink} = cGMP \times sink_c$	Delay state reactions<br><br>cGMP breakdown

3.1.3 Variables

Variable name	Variable unit	Variable value	Interpretation
k_{flow}	1/s	$kflow_{glucose} + stimulating - inhibiting$	Blood flow
G_{body}	amount	10	Glucose in arterial blood
O_{2body}	amount	10	Oxygen in arterial blood
oHb_{body}	amount	10	Oxygenated hemoglobin in arterial blood
dHb_{body}	amount	10	Deoxygenated hemoglobin in arterial blood
Stimulating	1/s	$b_1 \times cGMP + b_2 \times PG + b_3 \times EET$	Vasodilation
Inhibiting	1/s	$b_4 \times HETE$	Vasoconstriction
Act	1/s	$b_1 \times cGMP + b_2 \times PG + b_3 \times EET - b_4 \times HETE$	Signal substance effect on blood flow
ŷ	unitless	$\frac{k_y \times oHb}{dHb}$	Output signal

3.1.4 Parameters and parameter values

Parameter sets for model M_{nm1} used in Fig. 8, Fig. 9 and Fig. 10.

**Parameter names**	**Parameter unit**	**p7 best opt intensity data**	**p9 best opt frequency data**
k_y	amount/s	1223.4987	508.4496
$k_{metabolic}$	1/s	114.7037	1153.8404
$k_{neurotrans}$	1/s	1104.2209	116.0994
$k1_f$	1/s	177.3318	1174.2658
$k1_b$	1/(amount×s)	1122.5565	194.4418
k_{basal}	1/(amount×s)	91.3332	1381.4508
$kflow_{glucose}$	1/s	4.3346	117.9462
proportion1	oxygen/glucose metabolised	2.4188	5.6257
proportion2	oxygen/glucose metabolised	4.5876	2.6729
$sink_{Input}$	1/s	1.1802	4.6492
k_2	1/s	7.3947	1.1401
k_3	1/s	4.1432	3.7141
k_4	1/(amount×s)	2.0084	4.0534
k_5	amount/s	0.8885	2.1437
k_6	1/s	3.5817	0.9628
k_7	amount/s	0.3491	3.7232
k_8	unitless	1.0228	0.3676
k_9	unitless	0.1718	1.0583
k_{10}	1/s	5.8839	0.1795
k_{11}	1/s	49.7049	6.1628
k_{12}	1/s	0.5647	118.5220
k_{13}	1/s	0.9208	0.6189
k_{14}	1/s	485.3883	1.0366
k_{15}	1/s	3481.4269	583.7653
k_{16}	1/s	15170.2739	1937.3654
k_{17}	1/s	0.9036	10982.2259
k_{18}	1/s	1.0520	0.9339
k_{19}	1/s	1.1249	1.1366
k_{20}	1/s	350.7833	1.1654
k_{21}	1/s	1609.9603	592.9449
k_{22}	1/s	538.4678	1805.0249
k_{23}	1/s	8.8579	555.2464
km_1	amount	12.7144	9.2642
km_2	amount	7.8886	14.4421
Ca_{Abas}	amount/s	9279.9419	8.2545
Ca_{Nbas}	amount/s	1.5455	9828.1976
$sink_{Glu}$	1/s	0.9103	1.6419

$sink_A$	1/s	3.2162	0.8607
$sink_N$	1/s	0.6568	3.4898
$sink_H$	1/s	19985.5644	0.6677
$sink_P$	1/s	1.1195	19830.1832
$sink_E$	1/s	49.1725	1.1691
$sink_c$	1/s	3.8819	54.3174
NO_{sink}	1/s	37.9614	4.0331
PL	amount	40.8202	38.1518
b_1	1/(amount×s)	16.5399	40.5355
b_2	1/(amount×s)	18.6399	20.9122
b_3	1/(amount×s)	4.8399	19.3131
b_4	1/(amount×s)	1223.4987	5.0147

Cost: p7 = 7.17, p9 = 11.24.

3.1.5 Glucose metabolism in the model M_{nm1}

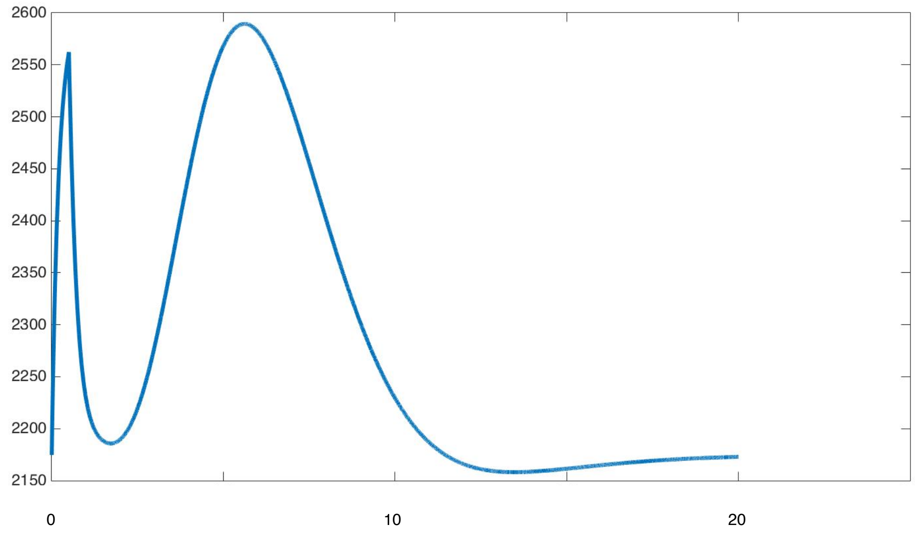

Figure G: Simulated glucose metabolism in the model M_{nm1}.

3.2 M_{nm2} Model structure

M_{nm1} is a minimized version of the model structure M_{nm1}.

3.2.1 Interactiongraph

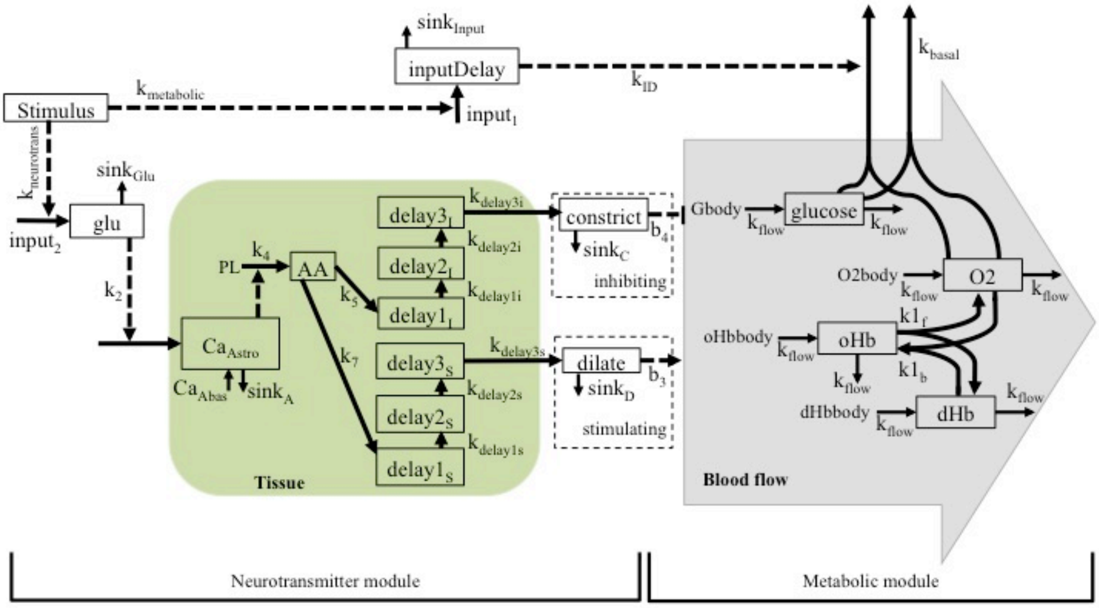

Fig. H: Interaction graph of the final model structure M_{nm2}. The model structure has two modules: the neurotransmitter module, which controls the blood flow, and the metabolic module, which controls the oxygen and glucose metabolism. Whole squares = states, dashed squares = variables (dependent on states), whole arrows = transformations, dashed arrows = interactions, green area = astrocyte, blue area = neuron, grey area = blood. All states starting with "delay" and a number (e.g delay2_S) are delay states. Stimulus is the input to the model. Stimulus = input signal. oHb and dHb are oxyhemoglobin and deoxyhemoglobin, respectively. Glu = glucose, Calcium neuron and calcium astrocyte = calcium ion (Ca^{2+}) level in the cell, AA = arachidonic acid. All terms starting with k (e.g k1), are parameters and in most cases represent rate constants. PL is a parameter representing phospholipase A_2, which is present in abundance. Gbody, O2body, oHbbody and dHbbody, are variables representing the glucose, oxygen and hemoglobin delivered into the area. "Constrict" and "dilate" are states representing the vasoactive substances which control the blood flow.

3.2.2 States and reactions

State	Interpretation	Steady state value: p8 best opt intensity	Steady state value: p10 best opt frequency
$\frac{d(stimulus)}{dt} = 0$	Stimulus input signal	1	1
$\frac{d(oHb)}{dt} = v1_b - v1_f + v_{inoHb} - v_{outoHb}$	Change in oxyhemoglobin level	3.76	3.28
$\frac{d(dHb)}{dt} = v1_f - v1_b + v_{indHb} - v_{outdHb}$	Change in deoxyhemoglobin level	16.24	16.72
$\frac{d(O_2)}{dt} = v1_f - v1_b - v_{basal} \times proportion_1 - v_{stim} \times proportion_2 + v_{inO_2} - v_{outO_2}$	Change in oxygen level	0.77	0.71
$\frac{d(glucose)}{dt} = v_{inG} - v_{outG} - v_{basal} - v_{stim}$	Change in glucose level	8.19	8.14
$\frac{d(inputDelay)}{dt} = input_1 - sink_{Input}$	Delay state	-4.86×10^{-15}	-1.00×10^{-16}
$\frac{d(glu)}{dt} = input_2 - glu_{sink}$	Glutamate release in the synaptic cleft	-2.53×10^{-17}	-6.09×10^{-19}
$\frac{d(Ca_{Astro})}{dt} = Glutamate_A - calcium_{Astro1} + Ca_{Abas}$	Calcium influx in the astrocyte	27.71	10.09
$\frac{d(AA)}{dt} = calcium_{Astro2} - (AA_i + AA_s)$	Change in AA level	386.51	124.27
$\frac{d(constrict)}{dt} = delay3_c - sink_C$<br>$\frac{d(delay1_i)}{dt} = AA_i - delay1_c$<br>$\frac{d(delay2_i)}{dt} = delay1_c - delay2_c$<br>$\frac{d(delay3_i)}{dt} = delay2_c - delay3_c$	Delay states	472.80<br>50.49<br>540.77<br>814.2	91.66<br>10.79<br>150.93<br>179.00
$\frac{d(dilate)}{dt} = delay3_d - sink_D$<br>$\frac{d(delay1_s)}{dt} = AA_s - delay1_d$<br>$\frac{d(delay2_s)}{dt} = delay1_d - delay2_d$<br>$\frac{d(delay3_s)}{dt} = delay2_d - delay3_d$	Delay states	210.92<br>455.61<br>280.27<br>216.58	96.019<br>143.60<br>135.65<br>57.206

Reactions	Interpretation
$v1_f = k1_f \times oHb$<br><br>$v1_b = k1_b \times dHb \times O_2$	Rate of releasing oxyhemoglobin into oxygen and deoxyhemoglobin<br><br>Rate of binding oxygen and deoxyhemoglobin into oxyhemoglobin
$v_{inoHb} = oHb_{body} \times k_{flow}$<br>$v_{outoHb} = oHb \times k_{flow}$<br><br>$v_{indHb} = dHb_{body} \times k_{flow}$<br>$v_{outdHb} = dHb \times k_{flow}$<br><br>$v_{inG} = G_{body} \times k_{flow}$<br>$v_{outG} = glucose \times k_{flow}$<br><br>$v_{inO_2} = O_{2body} \times k_{flow}$<br>$v_{outO_2} = O_2 \times k_{flow}$	Oxyhemoglobin influx<br>Oxyhemoglobin outflux<br><br>Deoxyhemoglobin influx<br>Deoxyhemoglobin outflux<br><br>Glucose influx<br>Glucose outflux<br><br>Oxygen influx<br>Oxygen outflux
$v_{basal} = k_{basal} \times O_2^{proportion_1} \times glucose$<br>$v_{stim} = inputDelay \times O_2^{proportion_2} \times glucose$	Basal metabolism<br>Metabolism during stimulation
$input_1 = k_{metabolic} \times stimulus$<br><br>$input_2 = k_{neurotrans} \times stimulus$	Stimulus input to the metabolic module<br>Stimulus input to the neurotransmitter module
$glu_{Sink} = glu \times sink_{Glu}$<br>$Glutamate_A = k_2 \times glu$<br>$calcium_{Astro1} = Ca_{Astro} \times sink_A$<br>$calcium_{Astro2} = PL \times Ca_{Astro} \times k_4$<br><br>$sink_C = constrict \times sink_{Con}$<br><br>$sink_D = dilate \times sink_{Dil}$<br><br>$AA_i = k_5 \times AA$<br><br>$AA_s = k_7 \times AA$	Glutamate breakdown<br>Calcium influx into the cells<br>Calcium outflux from the cells<br>Calcium inducing AA<br><br>Breakdown of vasoconstricting substances<br>Breakdown of vasodilating substances<br>AA triggering vasoconstricting substances<br>AA triggering vasodilating substances
$delay1_d = k_{delay1s} \times delay1_s$<br>$delay2_d = k_{delay2s} \times delay2_s$<br>$delay3_d = k_{delay3s} \times delay3_s$	Delay states
$delay1_c = k_{delay1i} \times delay1_i$<br>$delay2_c = k_{delay2i} \times delay2_i$<br>$delay3_c = k_{delay3i} \times delay3_i$	Delay states

3.2.3 Variables

Variable name	Variable unit	Variable value	Interpretation
k_{flow}	1/s	$kflow_{O2} + stimulating - inhibiting$	Blood flow
G_{body}	amount	10	Glucose in arterial blood
O_{2body}	amount	10	Oxygen in arterial blood
oHb_{body}	amount	10	Oxygenated hemoglobin in arterial blood
dHb_{body}	amount	10	Deoxygenated hemoglobin in arterial blood
Stimulating	1/s	$b_1 \times$dilate	Vasodilation
Inhibiting	1/s	$b_2 \times$constrict	Vasoconstriction
$\hat{y}$	unitless	$\frac{k_y \times oHb}{dHb}$	Output signal

3.2.4 Parameters and parameter values

Parameter sets for model M_{nm1} used in Fig. 9 and Fig. 10.

**Parameter names**	**Parameter unit**	**p8 best opt intensity data**	**p10 best opt frequency data**
k_y	unitless	3327.7279	3177.2096
$k_{metabolic}$	1/s	2425.3854	13592.202
$k_{neurotrans}$	1/s	140.35	195.9126
$k1_f$	1/s	2289.8029	6996.381
$k1_b$	1/(amount×s)	38.0878	72.7303
k_{basal}	1/(amount×s)	4751.4719	8097.4782
$kflow_{glucose}$	amount/s	524.89	1275.8673
proportion1	oxygen/glucose metabolised	8.6087	8.5580
proportion2	oxygen/glucose metabolised	3.8694	3.8495
$sink_{Input}$	1/s	0.6735	4.5253
k_2	1/s	0.4703	0.7295
k_4	1/(amount×s)	0.9977	1.2737
k_5	1/s	0.5446	0.7809
k_7	1/s	0.6699	0.5886
Ca_{Abas}	1/s	14.8381	20.5285
$sink_{Glu}$	1/s	6.4284	12.5502
$sink_A$	1/s	1.4713	0.7409
$sink_{Con}$	1/s	0.7384	0.6384
$sink_{Dil}$	1/s	1.4552	1.0505
kdelay1s	1/s	0.867	1.0787
kdelay2s	1/s	0.5797	0.4994
kdelay3s	1/s	0.6137	0.81178
kdelay1i	1/s	6.2735	5.9784
kdelay2i	1/s	0.4484	0.5582
kdelay3i	1/s	0.3781	0.3707
PL	amount	15	15
b_1	1/(amount×s)	30.379	41.1749
b_2	1/(amount×s)	13.2335	12.9425

Cost: p8 = 27.5 and p10 = 18.8
